# Enhanced Interaction between Pseudokinase and Kinase Domains in Gcn2 stimulates eIF2α Phosphorylation in Starved Cells

**DOI:** 10.1371/journal.pgen.1004326

**Published:** 2014-05-08

**Authors:** Sebastien Lageix, Stefan Rothenburg, Thomas E. Dever, Alan G. Hinnebusch

**Affiliations:** 1 Laboratory of Gene Regulation and Development, Eunice K. Shriver National Institute of Child Health and Human Development, National Institutes of Health, Bethesda, Maryland, United States of America; 2 Kansas State University, Division of Biology, Manhattan, Kansas, United States of America; University of Manchester, United Kingdom

## Abstract

The stress-activated protein kinase Gcn2 regulates protein synthesis by phosphorylation of translation initiation factor eIF2α, from yeast to mammals. The Gcn2 kinase domain (KD) is inherently inactive and requires allosteric stimulation by adjoining regulatory domains. Gcn2 contains a pseudokinase domain (YKD) required for high-level eIF2α phosphorylation in amino acid starved yeast cells; however, the role of the YKD in KD activation was unknown. We isolated substitutions of evolutionarily conserved YKD amino acids that impair Gcn2 activation without reducing binding of the activating ligand, uncharged tRNA, to the histidyl-tRNA synthetase-related domain of Gcn2. Several such Gcn^−^ substitutions cluster in predicted helices E and I (αE and αI) of the YKD. We also identified Gcd^−^ substitutions, evoking constitutive activation of Gcn2, mapping in αI of the YKD. Interestingly, αI Gcd^−^ substitutions enhance YKD-KD interactions in vitro, whereas Gcn^−^ substitutions in αE and αI suppress both this effect and the constitutive activation of Gcn2 conferred by YKD Gcd^−^ substitutions. These findings indicate that the YKD interacts directly with the KD for activation of kinase function and identify likely sites of direct YKD-KD contact. We propose that tRNA binding to the HisRS domain evokes a conformational change that increases access of the YKD to sites of allosteric activation in the adjoining KD.

## Author Summary

The survival of all living organisms depends on their capacity to adapt their gene expression program to variations in the environment. When subjected to various stresses, eukaryotic cells down-regulate general protein synthesis by phosphorylation of eukaryotic translation initiation factor 2 alpha (eIF2α). The yeast *Saccharomyces cerevisiae* has a single eIF2α kinase, Gcn2, activated by uncharged tRNAs accumulating in amino acid starved cells, which bind to a regulatory domain homologous to histidyl-tRNA synthetase. Gcn2 also contains a degenerate, pseudokinase domain (YKD) of largely unknown function, juxtaposed to the authentic, functional kinase domain (KD). Our study demonstrates that direct interaction between the YKD and KD is essential for activation of Gcn2, and identifies likely KD-contact sites in the YKD that can be altered to either impair or constitutively activate kinase function. Our results provide the first functional insights into the regulatory role of the enigmatic YKD of Gcn2.

## Introduction

Eukaryotic cells harbor stress-activated protein kinases that down-regulate protein synthesis and simultaneously up-regulate transcriptional activators at the translational level. This dual response allows cells to reduce bulk protein synthesis while re-programming transcription to favor expression of gene products with functions in stress management. The key target of these kinases is Ser-51 of the α-subunit of translation initiation factor 2 (eIF2α). The eIF2 bound to GTP transfers methionyl-initiator tRNA to the 40S ribosomal subunit to produce the 43S preinitiation complex at the beginning of the translation initiation pathway. On subsequent recognition of the AUG codon in mRNA by initiator tRNA, the GTP is hydrolyzed and eIF2-GDP is released from the 40S subunit for recycling to eIF2-GTP by the guanine nucleotide exchange factor eIF2B. Ser-51 phosphorylation converts eIF2 into an inhibitor of eIF2B, reducing the concentration of eIF2-GTP and delaying new rounds of translation initiation. The reduced eIF2-GTP level stimulates translation of *GCN4* mRNA in yeast and *ATF4* mRNA in mammals, both encoding transcriptional activators of stress genes, by allowing 43S complexes to circumvent small open reading frames present in their mRNA leaders that would normally block initiation at the protein coding sequences for Gcn4/Atf4 [1], [2] (reviewed in [3]).

The four mammalian eIF2α kinases, PKR, HRI, PERK, and Gcn2, have conserved kinase domains (KDs) but unique regulatory regions that mediate activation by distinct stress signals. PKR is activated by dsRNA generated during virus infection, and represents a key component of the antiviral defense mechanism, whereas Gcn2 is activated by uncharged tRNA that accumulates in amino acid-starved cells and most likely other stress conditions. The ensuing induction of Gcn4 in yeast evokes transcriptional activation of nearly all amino acid biosynthetic enzymes subject to the general amino acid control with attendant up-regulation of amino acid biosynthesis (reviewed in [3]). Translational control by mammalian Gcn2 is important for lipid homeostasis under starvation conditions [4], in behavioral aversion to amino acid-deficient diets [5], and in learning and memory [6]. It has also been implicated in tumor cell survival, both innate and T-cell mediated immune responses, and DNA repair upon UV irradiation (reviewed in [7]).

Because eIF2α kinases act by inhibiting translation, their functions must be tightly regulated to allow high-level kinase activity only under appropriate stress conditions. We showed previously that the Gcn2 KD is intrinsically inert and depends on stimulatory interactions with adjacent domains in the protein to achieve an active conformation [8]. This latency of Gcn2 depends on a rigid hinge connecting the N- and C-lobes, which promotes a partially closed active site cleft and occluded ATP-binding pocket, and a non-productive orientation of helix αC in the N-lobe that impedes proper disposition of a critical Lys reside that positions the ATP phosphates for catalysis [9], [10]. Binding of uncharged tRNA to a region C-terminal to the KD, related in sequence to the enzyme histidyl-tRNA synthetase (HisRS), which aminoacylates tRNA^His^, is required to activate Gcn2 in amino acid-starved cells [11], [12], [13], [14]. An N-terminal segment in the HisRS domain that interacts with a portion of the KD containing the hinge is required for kinase activation [15], suggesting that tRNA binding alters the HisRS-KD interface to evoke an active conformation of the KD.

As in other kinases, autophosphorylation of the activation loop of the KD is additionally required to activate Gcn2 [15], [16], as is dimerization of the KD [17] in a back-to-back orientation described for the active KD dimer of PKR [18]. The KD, HisRS region, and extreme C-terminal domain of Gcn2 (CTD) are capable of self-interaction as isolated domains; however, only the CTD is essential for dimerization and attendant activation of full-length Gcn2 [19], [20]. Since the HisRS-related domain and attendant tRNA-binding by Gcn2 are dispensable for dimerization, Gcn2 likely dimerizes constitutively through CTD self-interaction [19]. It is possible that the mode of KD dimerization switches from the antiparallel orientation seen in the crystal structure of the Gcn2 KD in an inactive conformation [9] to the parallel, PKR-like mode of dimerization deduced from genetic experiments [17] to represent the active conformation for Gcn2 [18].

In addition to dimerization, the CTD mediates ribosome association of Gcn2 [21], which is critical for activation of Gcn2 by uncharged tRNA in vivo [22]. The CTD also appears to interact with the KD in a manner that impedes kinase activation [14], [15], suggesting that dissociation of the CTD from the KD is a key step in the kinase activation pathway. The CTD further mediates an interaction with translation elongation factor eEF1A that appears to inhibit Gcn2 function in nonstarved cells and can be overcome by uncharged tRNA [23].

Activation of Gcn2 by uncharged tRNA additionally requires the functions of *trans-*acting factors Gcn1 and Gcn20, which form a complex that must interact with both the N-terminal “RWD” domain of Gcn2 and translating ribosomes to stimulate Gcn2 kinase function in yeast cells [24], [25], [26], [27], [28]. These findings, plus the fact that overexpression of translation elongation factor 3 impedes Gcn2 activation in vivo [29], support a model in which Gcn2 is activated by uncharged tRNA that binds first to the decoding center of a translating ribosome and is then transferred to the HisRS domain in Gcn2, and that Gcn1/Gcn20 stimulate one or both of these binding reactions involving uncharged tRNA [28].

Gcn2 contains a region N-terminal to the KD (aa291-538) that displays strong sequence similarity to authentic kinases, but lacks critical residues required for binding ATP and catalysis, and this “pseudokinase” domain (YKD) in mouse Gcn2 was found incapable of binding ATP or Mg^+2^ in vitro [30]. Studies of YKDs in other systems have indicated functions in regulating authentic KDs, as allosteric modulators of active KD conformation or as scaffolding molecules that promote assembly of higher-order kinase signaling complexes. Gcn2 and the Janus tyrosine kinase (JAK) family provide the only known instances where a YKD and KD reside in the same polypeptide. The YKD in the JAKs appears to maintain latency of the KD in the absence of cytokines; however, the molecular mechanism of YKD regulatory function is not well understood (reviewed in [30], [31]).

Elimination of the YKD from yeast Gcn2 abolishes activation of Gcn2 in amino acid starved cells and impairs the kinase activity of Gcn2 in vitro [13], [32] without affecting ribosome-binding [21], dimerization by full-length Gcn2 [19] or Gcn2 interaction with positive effectors Gcn1/Gcn20 [26]. Thus, the YKD seems to be required primarily for activation of the latent KD in Gcn2 by uncharged tRNA. The isolated yeast Gcn2 YKD can interact directly in vitro with the Gcn2 KD and CTD, and the YKD was shown to be required for high-level association of full-length Gcn2 with the isolated Gcn2 KD fused to LexA in vivo, presumably via YKD·LexA-KD interactions [19]. Hence, we hypothesized that the YKD allosterically activates Gcn2 via direct interaction with the KD.

To test this hypothesis rigorously, we have produced a structural model of the Gcn2 YKD based on its homology to authentic kinases, and made substitutions of residues predicted to be both surface-exposed and conserved among the YKDs of Gcn2 from different fungi. In this way, we identified (Gcn^−^) substitutions that impair Gcn2 activation under amino acid starvation conditions that, interestingly, appear to cluster on one face of the predicted tertiary structure of the YKD. We also conducted random mutagenesis of the YKD and identified (Gcd^−^) substitutions that confer constitutive activation of Gcn2 function and derepression of Gcn4 target genes involved in amino acid biosynthesis in vivo. Biochemical analysis of exemplar Gcn^−^ and Gcd^−^ substitutions provide strong evidence that the YKD directly interacts with the KD within the Gcn2 dimer to evoke allosteric activation of eIF2α kinase function in amino acid-starved cells, and the Gcn^−^/Gcd^−^ substitutions identify likely points of KD-YKD association in the YKD. Our results have important implications for the mechanism of Gcn2 activation by uncharged tRNA, and for the molecular functions of pseudokinases.

## Results

### Identification of Gcn^−^ substitutions in the YKD

**Figure 1:**
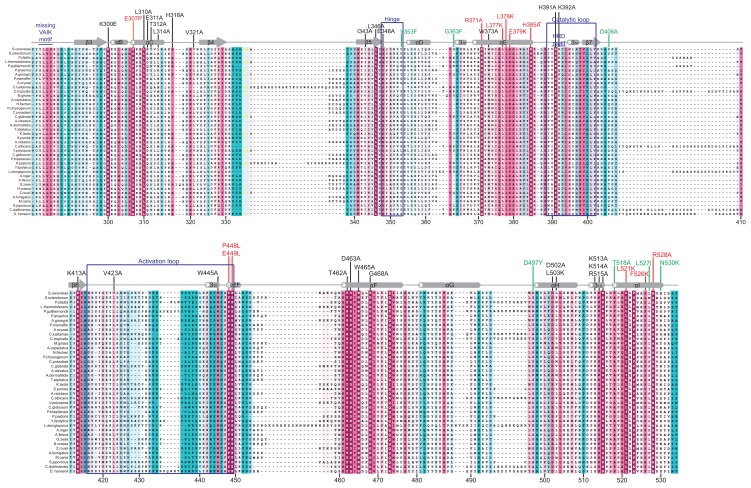
Structure-based sequence alignment of the YKD region of Gcn2. The multiple sequence alignment of YKDs from 40 fungal Gcn2 sequences, identified on the far left by abbreviations of their species of origin, was built using the MUSCLE program. Residues are colored according to evolutionary sequence variation as analyzed with the CONSURF on-line server, with magenta corresponding to the most conserved residues, and teal indicating the most variable. Numbering corresponds to residue positions in full-length *S. cerevisiae* Gcn2 (residues 280–534). Regions of predicted α-helical and β-strand secondary structures within the YKD are denoted above the *S. cerevisiae* sequence, based on the alignment of Gcn2 YKD sequences with authentic KDs in Fig. S1A–G. Substitutions (described below) conferring Gcn^−^ phenotypes are shown in red, those conferring Gcd^−^ phenotypes are shown in green, and those preserving WT function are shown in black.

To identify evolutionarily conserved residues and amino acids that are potentially critical for the regulatory function of the Gcn2 YKD, we constructed multiple sequence alignments of this domain using Gcn2 sequences from diverse fungi as well as the sequences of authentic KDs from Gcn2 and 11 other kinases (Fig. S1A–G). In accordance with previous alignments [30], [31], our analysis indicated that fungal Gcn2 YKDs lack critical features of authentic KDs, including the glycine-rich P-loop between the β1 and β2 strands, the “VAIK” motif (containing the critical Lys residue in β3 that positions ATP), and the “HRD” motif (containing the catalytic Asp) (Fig. S1A–G). They also lack the “DFG” motif in the activation loop, whose Asp residue promotes Mg^+2^ and ATP binding. As noted above, the mouse Gcn2 YKD was found to be incapable of binding ATP or Mg^+2^, supporting the conclusion that the Gcn2 YKD lacks kinase activity [30]. However, as shown in Fig. 1, there are numerous YKD segments highly conserved among fungal Gcn2 homologs, which likely include residues with important regulatory functions.

**Figure 2:**
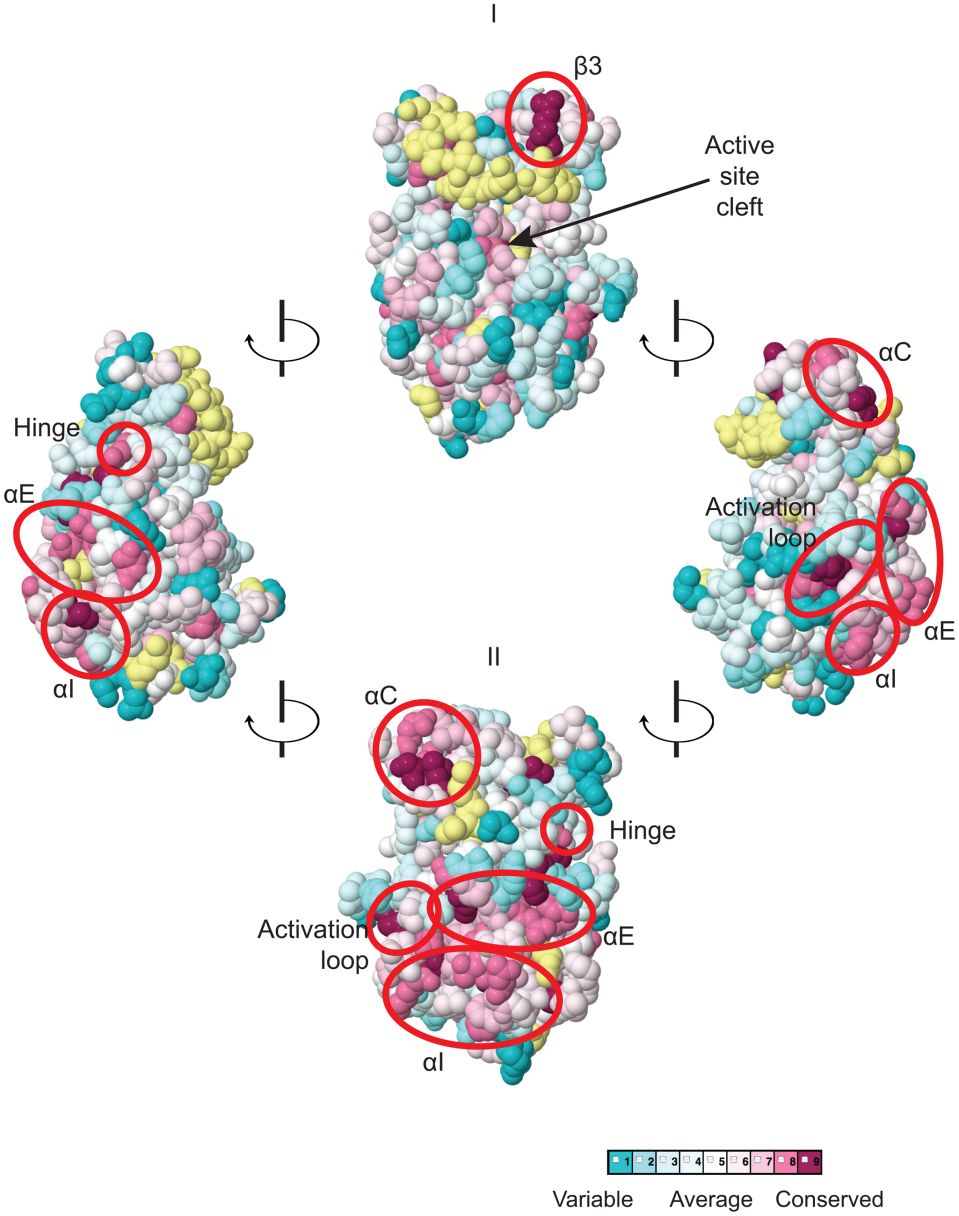
Predicted three-dimensional structure and sequence conservation of surface residues of the Gcn2 YKD. The degree of sequence conservation of Gcn2 YKD residues, shown in Fig. 1, was projected onto the three-dimensional structure of the authentic Gcn2 KD monomer using the CONSURF program. Yellow indicates amino acids for which the data are insufficient to calculate a reliable conservation grade. Except for residues in β3, the most highly conserved residues (magenta and red) are largely clustered on one surface (view II), whereas most of the variable residues (shades of blue) are on the opposite face (I). The most conserved regions (β3, αC, αE, αI, the activation loop and the hinge) are circled in red for emphasis.

We focused our mutagenesis experiments on conserved residues that, in most cases, are predicted to reside on the surface of the YKD and, hence, might contribute to its putative regulatory interactions with the KD in Gcn2. Because the structure of the YKD is unknown, we used our sequence alignment containing Gcn2 YKDs and authentic KDs (Fig. S1A–G) and projected sequence conservation for each YKD residue that could be aligned with a corresponding residue in authentic KDs onto the crystal structure of the authentic KD of *S. cerevisiae* Gcn2 [9]. The results (Fig. 2) predict that most highly conserved, surface-exposed residues occur in β3 and helix αC in the N-terminal lobe (N-lobe), in the hinge connecting the N- and C-terminal lobes, and in the activation loop and helices E and I in the C-terminal lobe. Except for β3 and αC, these conserved segments appear to comprise a largely contiguous surface on the “back-side” of the predicted C-lobe facing away from the “active site” cleft (Fig. 2, view II).

**Figure 3:**
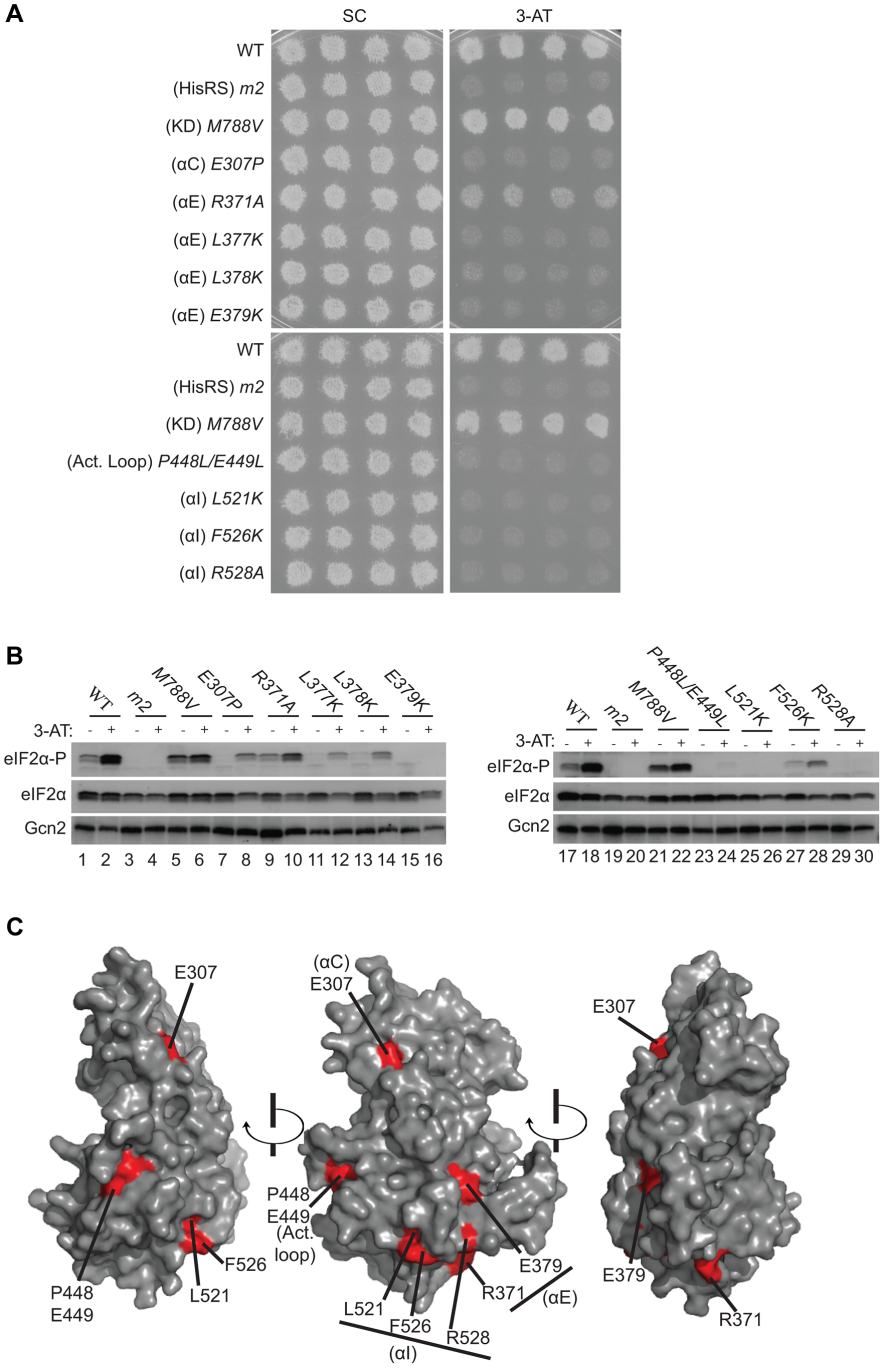
Substitutions of predicted surface-exposed residues of the Gcn2 YKD conferring Gcn^−^ phenotypes *in vivo*. (**A**) Transformants of *gcn2*Δ strain H1149 containing derivatives of low-copy plasmid p722 with WT *GCN2*, *gcn2-m2*, *GCN2^c^-M788V*, or the indicated mutations in the YKD were replica-plated to synthetic complete medium lacking uracil (SC-Ura) and SC-Ura plus 30 mM 3-AT and incubated for 3 d at 30°C. (**B**) Cultures of strains from panel A were grown in liquid SC medium lacking uracil and histidine to saturation, diluted into fresh medium at A_600_ of ∼0.2, and grown 6 h at 30°C. 3-AT was added at 10 mM to one culture for 1 h before harvesting (even-numbered lanes). WCEs were resolved by SDS-PAGE and subjected to Western analysis using the indicated specific antibodies and enhanced chemiluminescence to detect immune complexes. (**C**) Localization of the Gcn^−^ substitutions on the predicted 3-D structure of the Gcn2 YKD domain. Residues in the authentic Gcn2 KD that align in Fig. S1A–G with Gcn^−^ substitutions in the YKD from (A) were colored red and labeled on the crystal structure of the Gcn2 KD monomer.

To probe the regulatory functions of conserved YKD residues predicted to be surface-exposed in the structural model (Fig. 2), we used site-directed mutagenesis of *GCN2* on a single-copy (sc) plasmid to alter 43 such residues, generally making alanine substitutions or introducing a charged residue in place of a bulky hydrophobic residue or one of opposite charge. The resulting mutant *GCN2* alleles were tested for complementation of the 3-aminotriazole (3-AT) sensitivity of a (*gcn2*Δ) strain lacking chromosomal *GCN2*. 3-AT is an inhibitor of histidine biosynthesis that activates Gcn2, with attendant induction of *GCN4* translation, and the induced Gcn4 stimulates transcription of histidine (and other amino acid) biosynthetic enzymes in a manner required for growth in the presence of 3-AT. Thus, mutations that reduce Gcn2 activation confer 3-AT sensitivity (3-AT^S^), as illustrated in Fig. 3A (row 2) for the double substitution in the HisRS-like domain (Y1119L/R1120L) encoded by the *gcn2*-*m2* allele, which impairs tRNA binding [12], [14]. By contrast, the *GCN2^c^-M788V* allele, conferring constitutive activation of Gcn2 [33], supports strong growth on 3-AT comparable to that of wild-type (WT) *GCN2* (Fig. 3A, row 3).

Most mutations we examined did not detectably affect Gcn2 function, conferring no reduction in growth on 3-AT medium (Fig. S2A–B and data not shown; summarized in Fig. S2C). However, we identified several substitutions that conferred 3-AT^S^ phenotypes comparable to that of *m2*, indicating strong Gcn^−^ phenotypes (Fig. 3A and data not shown; summarized as red substitutions in Fig. 1 and listed in Fig. S2C). These Gcn^−^ mutations include substitutions of a residue at the beginning of helix αC (E307P), substitutions of 5 residues in predicted αE (R371A, L377K, L378K, E379K and H385A), a double substitution at the C-terminal end of the predicted activation loop (P448L/E449L), and 3 substitutions in the predicted C-terminal helix αI (L521K, F526K, and R528A). (Henceforth, for simplicity, we will refer to secondary structure elements of the YKD without stipulating in every instance that they are hypothetical predictions of the model in Fig. 2.)

Consistent with their strong 3-AT^S^ phenotypes, the Gcn^−^ YKD substitutions impaired eIF2α phosphorylation by Gcn2 in vivo. Western analysis of whole cell extracts (WCEs) from WT cells revealed that 3-AT evokes the expected increase in eIF2α phosphorylated on Ser-51 (eIF2α-P) relative to total eIF2α, whereas *m2* cells have no detectable eIF2α-P; and *M788V* cells display high-level eIF2α-P with or without 3-AT treatment (Fig. 3B, lanes 1–6 & 17–22; and data not shown). Importantly, except for R371A and H385A, all of the Gcn^−^ YKD substitutions greatly reduce or abolish eIF2α-P both in non-starvation conditions and in 3AT-treated cells, without producing a noticeable reduction in Gcn2 abundance (Fig. 3B, lanes 7–16 & 23–30). Consistent with its leaky 3-AT^S^ growth phenotype, the *R371A* mutation confers only a moderate reduction in eIF2α-P in 3AT-treated cells (lanes 1–2 vs. 9–10). The *H385A* allele was eliminated from consideration because it produced no detectable Gcn2 (data not shown; Fig. S2C). Thus, as summarized in Fig. 3C, conserved surface residues in predicted helices C, E, and I, and in the activation loop of the YKD are required for WT activation of Gcn2 in vivo. These residues might mediate an important regulatory interaction between the YKD and the KD that overcomes the latency of KD function in response to amino acid starvation. (Two of the Gcn^−^ substitutions in helix αE, L377K and L378K, alter residues predicted to be buried in the YKD and thus might disrupt αE rather than eliminating a specific contact involving the YKD; hence, E379K was chosen as the exemplar αE substitution for subsequent analyses below.)

### Identification of Gcd^−^ substitutions in the YKD

**Figure 4:**
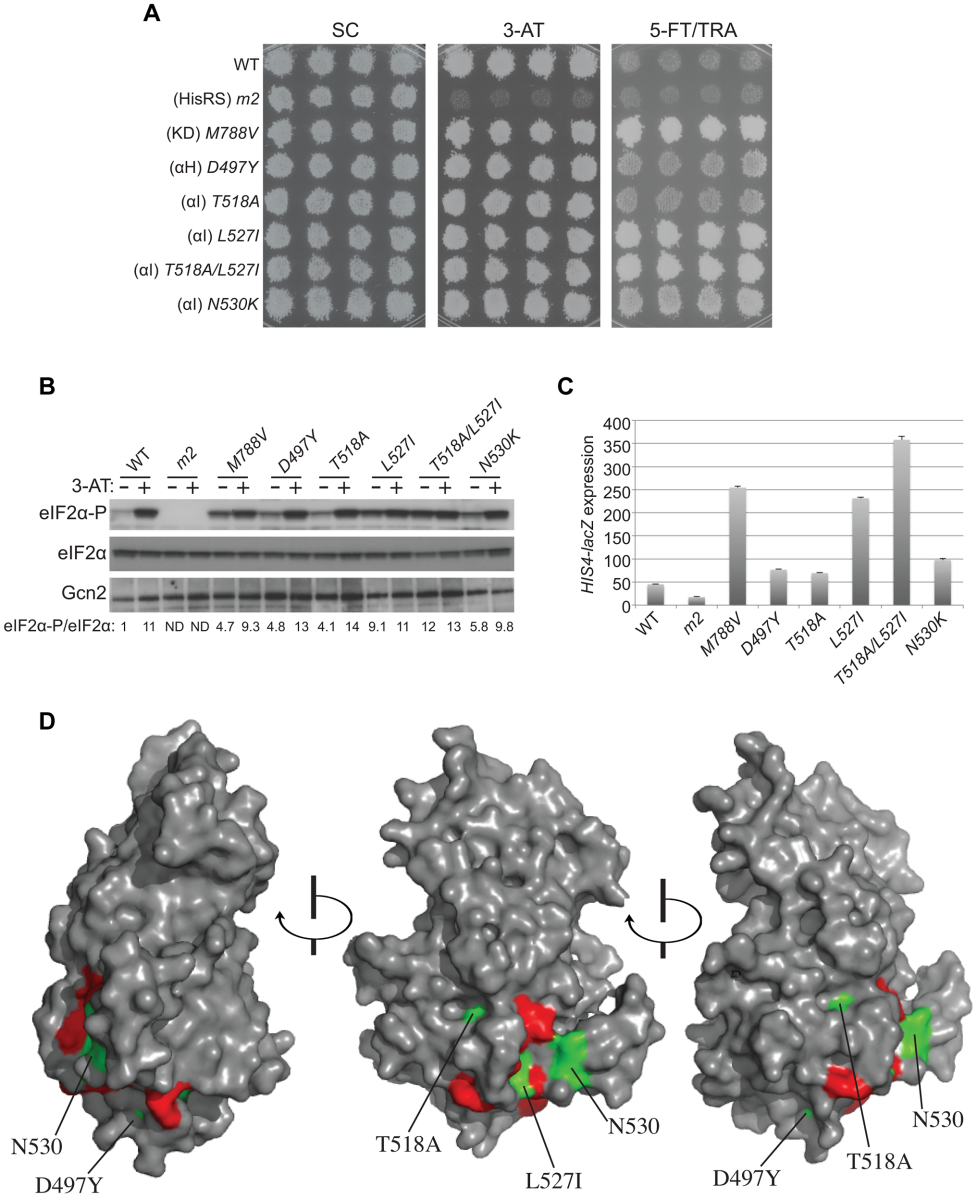
Mutations in the αI helix of the YKD constitutively activates Gcn2 *in vivo*. (**A**) Transformants of *gcn2*Δ strain H1149 containing p722 derivatives with WT *GCN2*, *gcn2-m2*, *GCN2^c^-M788V*, or mutations affecting residues in helix αI of the YKD were replica-plated to SC-Ura, SC-Ura plus 30 mM 3-AT, or SD plus 0.5 mM 5-FT and 0.125 mM TRA (5FT/TRA) and incubated for 3 d at 30°C. (**B**) Cultures of strains from panel A were analyzed for levels of eIF2α-P as in Fig. 3B. Western signals on the upper panel (P-eIF2α) were quantified by scanning densitometry of exposed films using ImageJ software, normalized for the corresponding signals in the middle panel (total eIF2α), and the ratios of the two signals (eIF2α-P; eIF2α) are indicated below the lanes. Standard errors are less than 6.5% of the mean values shown. (**C**) Gcd^−^ phenotypes of the indicated mutants were quantified by measuring *HIS4-lacZ* expression. Strains from (A) were cultured in nonstarvation conditions as described in Materials and Methods and WCEs were prepared and assayed for β-galactosidase activities. Results are the means and S.E.M.s calculated from three transformants, with activity expressed as nanomoles of *o*-nitrophenyl-β-D-galactopyronoside hydrolyzed per minute per milligram of protein. (**D**) Locations on the predicted structure of the YKD domain of Gcd^−^ substitutions (from panel A; green) and a subset of Gcn^−^ substitutions in αE and αI (red) determined as in Fig. 3C.

We reasoned that if the positive regulatory function of the YKD is modulated by amino acid availability, it should be possible to obtain *GCN2^c^* mutations mapping in the YKD that constitutively activate Gcn2 function. To test this prediction, we randomly mutagenized the YKD coding sequences in *GCN2*, introduced a library of mutant plasmids into the *gcn2*Δ strain and selected for clones growing on medium containing tryptophan analog 5-fluorotryptophan (5-FT) and histidine analog triazolealanine (TRA). Resistance to both 5-FT and TRA (5FT^R^/TRA^R^) results from Gcn4-mediated derepression of tryptophan and histidine biosynthetic enzymes in nonstarvation conditions, diminishing the toxic effects of 5-FT/TRA on protein synthesis, and is a sensitive indicator of constitutive activation of Gcn2 [33]. Accordingly, *GCN2^c^* mutations, such as *M788V*, confer growth on 5-FT/TRA medium, whereas *GCN2^+^* cells (and Gcn^−^ strains like *gcn2-m2*) are sensitive to the analogs (5-FT^S^/TRA^S^) (Fig. 4A, rows 1–3).

By screening the mutagenized plasmid library, we identified three mutations conferring growth on 5-FT/TRA medium that alter residues located within, or just C-terminal to, helix αI (Fig. 1, green substitutions). *T518A*, mapping in the N-terminus of αI, confers a mild 5-FT^R^/TRA^R^ phenotype, whereas *L527I* and *N530K*, mapping within or just C-terminal to αI, confer stronger analog-resistance phenotypes, which for *L527I* and the double substitution *T518A/L527I* are equivalent to that of *GCN2^c^-M788V* (Fig. 4A). We also identified a mutation just N-terminal to predicted αH, *D497Y*, with a mild 5-FT^R^/TRA^R^ phenotype similar to that of *T518A*. Importantly, these mutations elevate eIF2α-P under nonstarvation conditions to an extent commensurate with their 5-FT^R^/TRA^R^ phenotypes, as the mutations conferring the strongest 5-FT^R^/TRA^R^ phenotypes, *L527I* and *T518A/L527I*, also evoke the largest eIF2α-P/eIF2α ratios in cells grown without 3-AT (Fig. 4B).

These YKD mutations also derepress expression of a Gcn4-dependent *HIS4-lacZ* reporter in nonstarvation conditions, thus confirming their Gcd^−^ phenotypes. As expected, WT cells express this reporter at low levels in non-starvation conditions, whereas *GCN2^c^-M788V* cells display ∼5-fold higher levels of reporter expression. The YKD mutations elevate *HIS4-lacZ* expression to an extent that parallels their 5-FT^R^/TRA^R^ phenotypes, with *D497Y* and *T518A* conferring only ∼150% increases, *N530K* and *L527I* conferring ∼2-fold and ∼5-fold increases, respectively, relative to WT, and *T518A/L527I* exceeding the effect of *GCN2^c^-M788V* (Fig. 4C). Hence, these four YKD mutations are *bona fide GCN2^c^* alleles that activate Gcn2 in non-starvation conditions. Interestingly, they alter surface-exposed residues, with the two mutations with strongest Gcd^−^ phenotypes, *N530K* and *L527I*, altering residues predicted to have the greatest exposure (among the Gcd^−^ substitutions) and to reside in proximity to one another (Fig. 4D). It is also intriguing that both Gcn^−^ and Gcd^−^ substitutions were identified in helix αI, in one case (F526K and L527I) substituting adjacent residues with opposite outcomes for Gcn2 function (Fig. 4D), thus underscoring the importance of αI in regulating Gcn2 function.

**Figure 5:**
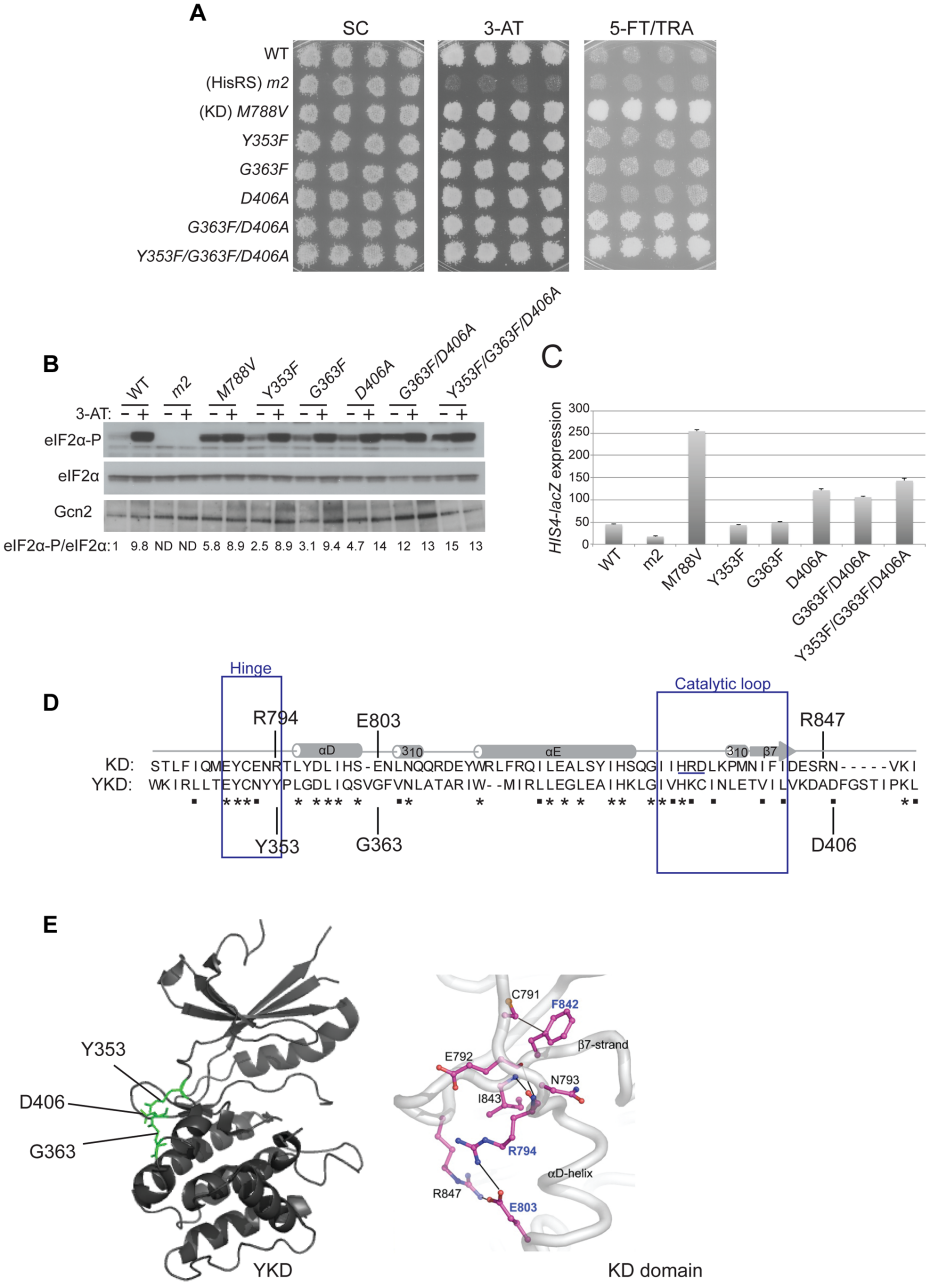
Substitutions in the predicted hinge of the YKD constitutively activate Gcn2 *in vivo*. (**A**) Transformants of *gcn2*Δ strain H1149 harboring the indicated *GCN2* alleles were analyzed for resistance to 3-AT and 5-FT/TRA as in Fig. 4A. (**B**) Strains from (A) were analyzed for eIF2α-P as in Fig. 4B. Standard errors are less than 8.5% of the mean values shown. (**C**) Strains from (A) were analyzed for *HIS4-lacZ* expression in non-starvation conditions as in Fig. 4C. (**D**) Structure-based sequence alignment and conservation of the hinge regions of the Gcn2 KD and YKD domains. Regions of predicted α-helical and β-strand secondary structures are denoted schematically above sequence. The HRD catalytic motif in the KD domain is underlined. Mutations causing Gcd^−^ phenotypes in each domain are indicated. (**E**) Locations of Gcd^−^ substitutions on the predicted YKD structure (left) and KD (right, in boldface) along with the residue interactions that rigidify the hinge in the KD domain (right). (Image on the right reproduced from Fig. 3C of Padyana et al. (2005)).

Three other *GCN2^c^* mutations were identified in our screen that alter residues located near the predicted hinge connecting the N- and C-lobes of the YKD. These include *Y353F*, mapping between β5 and αD in the hinge itself, *G363F* mapping between αD and αE, and *D406A* mapping between β7 and β8 (Fig. 1). Although individually they confer only slight increases in growth on 5-FT/TRA medium, stronger 5-FT^R^/TRA^R^ phenotypes were produced by the combination of *G363F* and *D406A*, or of all three mutations, in the same allele, which was achieved by site-directed mutagenesis (Fig. 5A). Moreover, whereas the single mutations evoked relatively small increases in basal eIF2α-P, the double and triple mutants conferred relatively larger increases in eIF2α-P under nonstarvation conditions compared to WT cells (Fig. 5B). The double and triple YKD mutations also derepressed the *HIS4-lacZ* fusion in non-starvation conditions, conferring Gcd^−^ phenotypes (Fig. 5C). Thus, *G363F/D406A* and *Y353F/G363F/D406A* are additional *GCN2^c^* alleles that activate Gcn2 in non-starvation conditions.

It is intriguing that an alignment of the YKD and authentic KD of yeast Gcn2 reveals that the YKD residues substituted by *Y353F*, *G363F*, and *D406A* align closely with KD residues, R794, E803 and R847, respectively (Fig. 5D), which interact with one another and rigidify the hinge of the KD [9] (Fig. 5E). Two of these KD residues (R794 and E803) are altered by *GCN2^c^* mutations [8], [33], leading to the model that hinge rigidity contributes to latency of the Gcn2 KD by impeding inter-lobe mobility [9]. It is intriguing to consider the possibility that the Y353F and D406A substitutions could eliminate hydrogen bonding and salt-bridge interactions, respectively, and that G363F could perturb the orientation of a nearby residue in αD, which all could normally promote hinge rigidity of the YKD. In this event, increasing the flexibility of the predicted hinge and interlobe mobility in the YKD could be responsible for the ability of these substitutions to activate the KD in the absence of high-level binding of uncharged tRNA to the HisRS domain and thereby confer the Gcd^−^ phenotype.

It is noteworthy that all of the Gcd^−^ variants harboring single substitutions in the YKD confer higher levels of eIF2α-P in histidine starved, 3-AT-treated cells compared to non-starved cells (eg., + and − lanes for D497Y and T518A in Fig. 4B). This observation suggests that the Gcd^−^ mutants retain the ability to bind uncharged tRNA and can be activated to a greater extent than WT Gcn2 by basal levels of uncharged tRNA in non-starved cells.

### YKD regulatory mutations alter Gcn2 kinase activity in vitro without commensurate changes in tRNA binding

Uncharged tRNA is the activating ligand for Gcn2, and there are Gcn^−^ and Gcd^−^ mutations known that impair or enhance tRNA binding, respectively, by purified Gcn2 in vitro [14], [34]. While there is no evidence that the YKD affects tRNA binding to the HisRS-like domain in Gcn2, this possibility could not be dismissed *a priori*. Accordingly, we examined whether exemplar Gcn^−^ and Gcd^−^ mutations in the YKD have the expected effects on Gcn2 kinase function in vitro, and whether these alterations in kinase activity are associated with corresponding changes in tRNA binding. Mutant and WT Gcn2 proteins were purified from yeast and tested for kinase activity using [^32^P]-labeled ATP and a recombinant yeast eIF2α peptide as substrates, and SDS-PAGE/autoradiography to detect the reaction products. It was shown previously that WT Gcn2 displays similar kinase activity whether purified from starved or non-starved cells and that the Gcn^−^ *m2* mutation, which impairs tRNA binding by Gcn2, reduces the kinase activity of purified Gcn2. This effect of the *m2* mutation was attributed to reduced activation of Gcn2 in vitro by deacylated tRNA present in cell lysates prior to Gcn2 purification [13].

**Figure 6:**
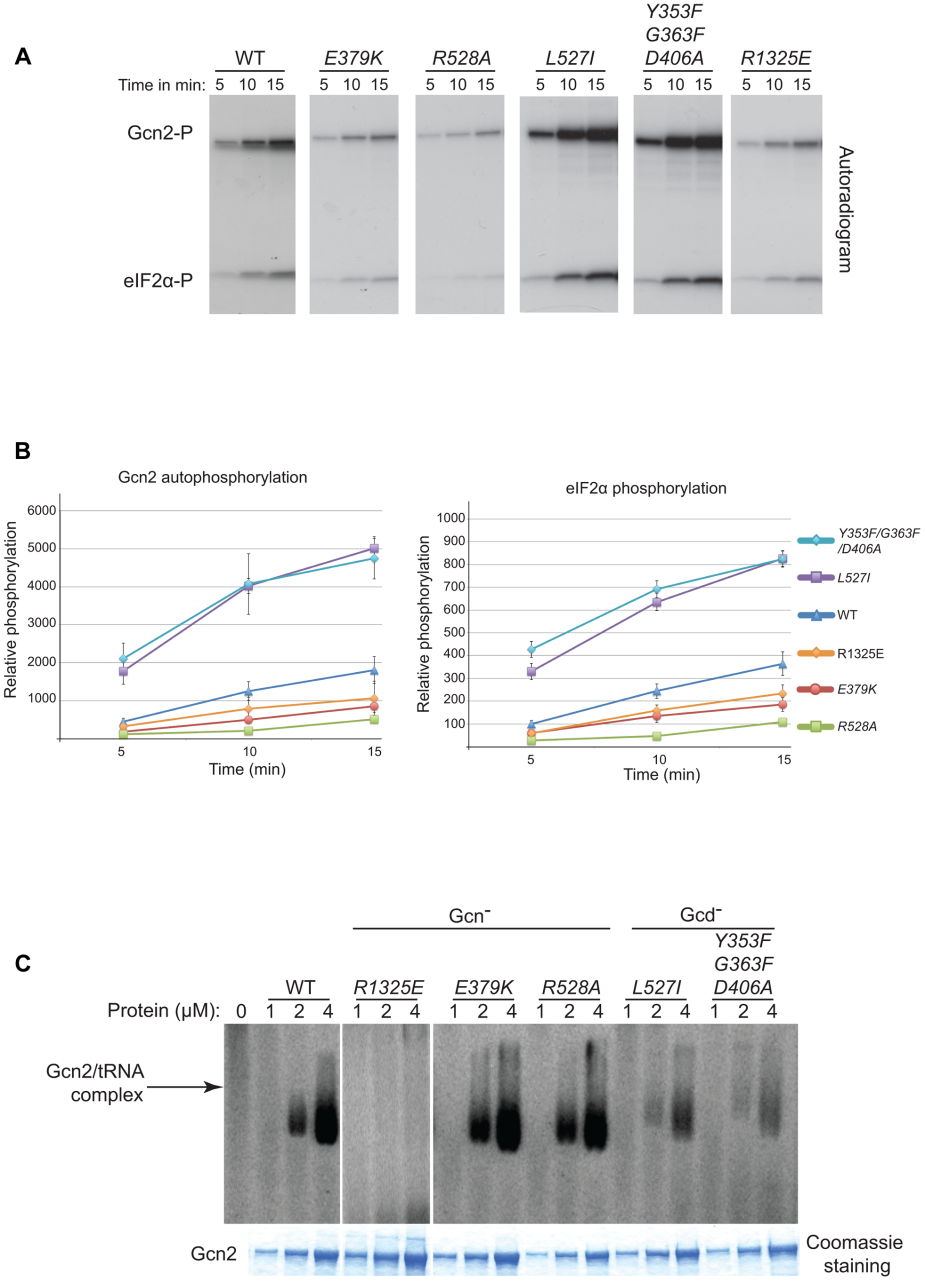
Effect of YKD substitutions on kinase activity and tRNA binding by purified Gcn2 *in vitro*. (**A**) The indicated Gcn2 proteins (0.25 µg) were incubated with 3 µCi of [γ-^32^P]ATP (6000 Ci/mmol, Amersham), 1 µg of recombinant eIF2α−ΔC purified from *E. coli*, and 0.5 µg of bovine serum albumin in 20 µL of kinase assay buffer (20 mM Tris–HCl [pH 7.9], 50 mM NaCl, 10 mM MgCl_2_, 1 mM dithiothreitol, and 100 µM PMSF) for 5 to 15 min at 30°C. The samples were resolved by 8%–16% SDS–PAGE and subjected to autoradiography. Positions of autophosphorylated Gcn2 (Gcn2-P) and phosphorylated eIF2α−ΔC (eIF2α-P) are indicated. All results were cropped from the same autoradiogram except for those obtained for the R1325E variant, which was analyzed separately with the same WT protein. The results for WT in the latter autoradiogram were essentially identical to those shown here. (**B**) The extent of Gcn2 autophosphorylation and eIF2α phosphorylation at each time point in (A) was determined by quantifying the intensity of the relevant bands by phosphorimaging of the respective gel bands. Data obtained from 3 independent experiments was averaged and plotted with S.E.M.s as error bars. (**C**) Purified Gcn2 proteins were incubated at the indicated concentrations with [^32^P]-labeled total yeast tRNA in 20 µL of GMSA buffer. Gcn2-tRNA complexes were resolved by electrophoresis through a 1% agarose gel in 1×MOPS buffer (1.5 h, 100 V), transferred to a nitrocellulose membrane and visualized by autoradiography. Unbound [^32^P]-tRNA, which has a higher mobility, was present at essentially identical amounts in each lane at levels ∼15-fold higher than the WT Gcn2/tRNA complexes formed at 4 µM (data not shown). All results shown originate from the same gel except for those obtained for the R1325E variant, which was analyzed separately with the same WT protein. The results for WT in the latter gel were similar to those shown here.

Consistent with previous findings [13], we observed that a Gcn2 mutant harboring a substitution in the HisRS domain (R1325E) that impairs tRNA binding in vitro (see below) reduces both the autophosphorylation and eIF2α kinase activity of purified Gcn2 (Fig. 6A–B). The YKD Gcn^−^ mutants E379K (αE substitution) and R528A (αI substitution) exhibit comparable (E379K) or even larger (R528A) reductions in both autophosphorylation and eIF2α phosphorylation compared to the R1327K variant. These findings are consistent with the conclusion that the YKD Gcn^−^ substitutions impair activation of Gcn2 by uncharged tRNA. By contrast, the Gcd^−^ mutants L527I (αI substitution) and Y353F/G363F/D406A (hinge-related substitutions) display substantially higher than WT observed rates of autophosphorylation and substrate phosphorylation (Fig. 6A–B), consistent with the idea that these substitutions increase the ability of the KD to be activated by uncharged tRNA.

To determine if the changes in kinase activity evoked by these mutations result from alterations in tRNA binding affinity, we tested the purified Gcn2 variants for binding of [^32^P]-labeled total tRNA using a gel mobility shift assay to detect Gcn2-tRNA complexes. The R1325E substitution in the HisRS domain eliminated detectable binding of uncharged tRNA by Gcn2 in vitro (Fig. 6C), consistent with its strong Gcn^−^ phenotype (S.L. and A.G.H., unpublished observations). By contrast, the Gcn^−^ mutants E379K and R528A exhibit tRNA binding indistinguishable from WT Gcn2 (Fig. 6C), implying that their inability to be activated in starved cells does not result from reduced binding of uncharged tRNA to the HisRS-like domain. Interestingly, the Gcd^−^ mutants L527I and Y353F/G363F/D406A show reduced tRNA binding activity (Fig. 6C), at odds with the possibility that the constitutive activation of kinase function displayed by these variants results from increased affinity for uncharged tRNA. One explanation for this last result could be that the reduced tRNA binding by the Gcd^−^ variants results from a putative negative autoregulation of tRNA binding in response to hyperactivation of kinase function. Another possibility would be that it reflects a greater than WT level of co-purification of the Gcd^−^ variants with endogenous tRNA. The latter explanation is very improbable in view of our finding that the purified Gcn2 preparations contain only small amounts of tRNA, estimated to be <5% on a molar basis, which do not vary between the WT, Gcd^−^ and Gcn^−^ proteins analyzed in Fig. 6 (data not shown).

### Evidence that YKD Gcd^−^ substitutions in αI enhance YKD-KD interactions

**Figure 7:**
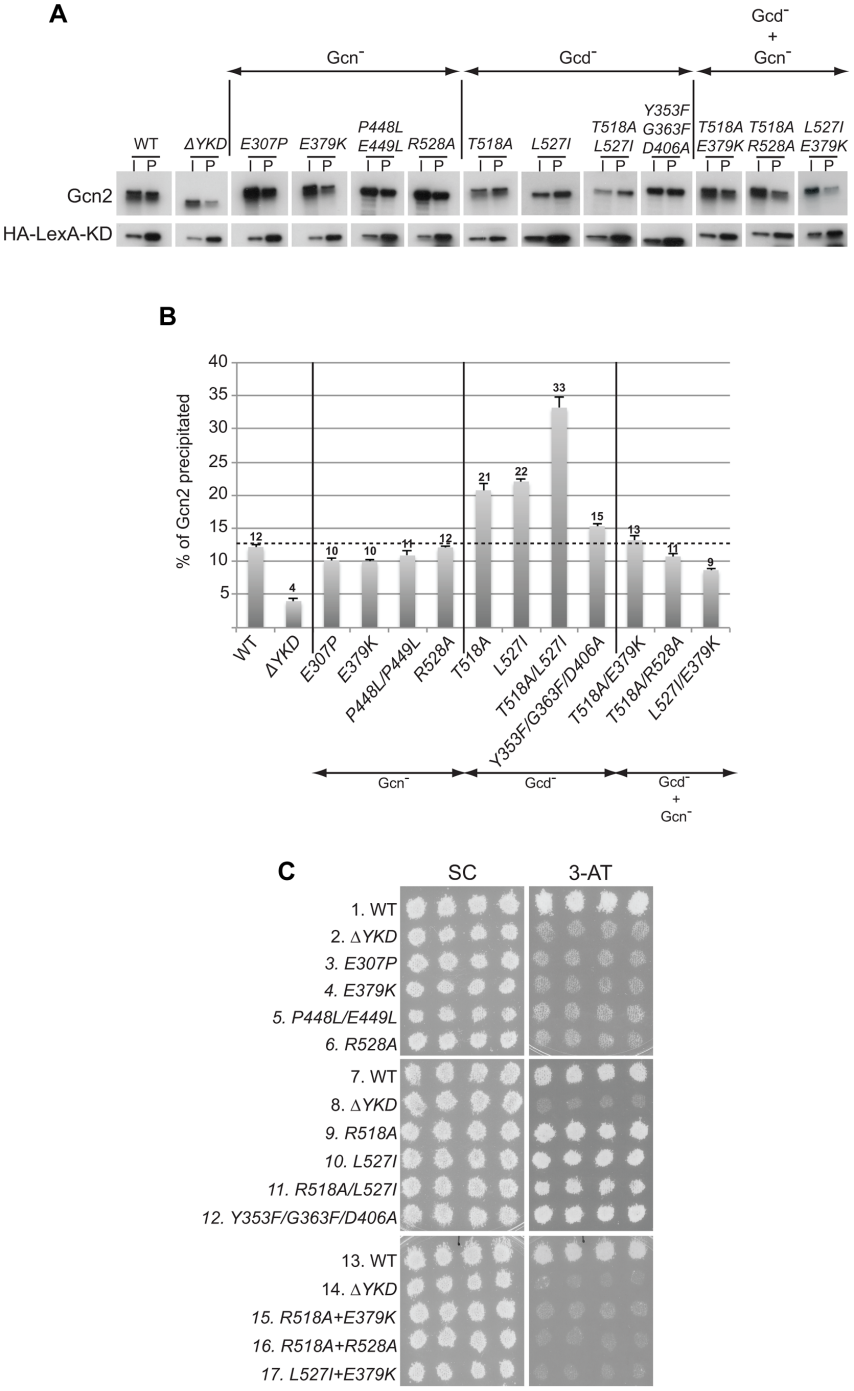
Certain Gcd^−^ substitutions in the YKD enhance coimmunoprecipitation of Gcn2 with LexA-HA-KD from yeast extracts. (**A**) WCEs were prepared from transformants of *gcn2*Δ strain HQY132 bearing high-copy-number plasmid p2825 encoding LexA-HA-KD (720–999) and plasmids encoding wild-type Gcn2 (p630) or the indicated Gcn2 mutant. Aliquots of extracts containing 50 µg of protein were immunoprecipitated with anti-HA antibodies and the precipitates were resolved by SDS-PAGE and subjected to immunoblot analysis with anti-Gcn2 antibodies (upper panel) or anti-LexA antibodies (lower panel), and enhanced chemiluminescence was used to detect immune complexes. Input (I) lanes contain 5 µg of starting WCEs and pellet (P) lanes contain immune complexes recovered from 25 µg of WCEs. All results shown for each protein were cropped from the same immunoblot. (**B**) Densities of bands in I and P lanes of (A) were quantified by scanning densitometry of exposed films using ImageJ software, and P∶I ratios of Gcn2 signals were normalized to the LexA-HA-KD signals in the corresponding P lanes. The normalized ratios were calculated from three independent experiments and the average and S.E.M.s were plotted for each Gcn2 variant. (**C**) Transformants of *gcn2*Δ strain HQY132 containing high-copy-number plasmids encoding WT Gcn2 (p630) or the indicated YKD mutants were replica-plated to SC-Ura and SC-Ura plus 30 mM 3-AT and incubated for 3 d at 30°C.

We next considered the possibility that the YKD mutations alter a regulatory interaction between the YKD and KD that evokes allosteric activation of Gcn2 kinase function. To address this possibility, we first employed an assay described previously wherein full-length Gcn2 (WT or YKD mutants) is coimmunoprecipitated from cell extracts with an HA epitope-tagged LexA fusion to a Gcn2 KD segment (residues 720–999) that harbors a portion of the large Gcn2-specific insert between strands β4 and β5, strand β5 from the N-lobe, the hinge, and entire C-lobe. While this represents an incomplete KD, the C-lobe can be expected to fold independently of the N-lobe [9]; and this KD segment was shown previously to interact specifically with the CTD in a manner dependent on the C-terminal portion of the large KD insert and impaired by the *GCN2^c^-E803V* Gcd^−^ substitution of a key residue in the KD region [15]. Moreover, in agreement with previous results, we found that deletion of the entire YKD reduces interaction of otherwise full-length Gcn2 with this HA-LexA-KD fusion. Thus, the proportion of Gcn2-ΔYKD present in the input (I) sample that co-immunoprecipitates with the HA-LexA-KD fusion in the pellet (P) fraction was decreased to ∼1/3^rd^ of the level seen with WT Gcn2 (Fig. 7A–B, WT vs. Δ*YKD*). This reduction has been attributed to loss of interaction between the YKD in full-length Gcn2 and the KD segment in HA-LexA-KD [19]. We found here that YKD Gcn^−^ substitutions in αC (E307P), αE (E379K), the activation loop (P448L/P449L) and αI (R528A), as well as the Gcd^−^ hinge-related triple substitutions Y353F/G363F/D406A, had little or no effect on the percentage of Gcn2 coimmunoprecipitated with HA-LexA-KD. By contrast, the Gcd^−^ substitutions in αI, T518A, L527I, and T518A/L527I, all reproducibly increased the proportion of the input Gcn2 recovered with HA-LexA-KD in the pellet (Fig. 7A–B). (Note that results in Fig. 7B were obtained by calculating the ratio of P to I signals for each variant and normalizing the P∶I ratios by the intensity of HA-LexA-KD in the P fraction for that variant.) It is noteworthy that the Gcn2-T518A/L527I double mutant showed a reproducibly higher level of coimmunoprecipitation with HA-LexA-KD compared to the corresponding two single mutants (Fig. 7B), commensurate with the relatively stronger Gcd^−^ phenotype of the double mutant (Fig. 4C).

Considering that the increases in Gcn2:HA-LexA-KD association provoked by the Gcd^−^ substitutions T518A and L527I were ≤33% (Fig. 7B), we sought additional evidence that these results are physiologically relevant to the increased kinase function evoked by the Gcd^−^ substitutions. To this end, we evaluated the effects of double substitutions combining these two Gcd^−^ substitutions with Gcn^−^ substitutions E379K and/or R528A. Interestingly, all three double mutants we examined exhibit 3-AT^S^ and 5-FT^S^/TRA^S^ phenotypes indistinguishable from the Gcn^−^ single mutants (Fig. 7C, rows 15–17 vs. 9–10, 4 & 6; and data not shown), indicating that the Gcn^−^ substitutions fully suppress the activating effects of the Gcd^−^ substitutions. We reasoned that if the tighter association between the YKD and KD contributes to the Gcd^−^ phenotypes of T518A and L527I, then the Gcn^−^ substitutions should also suppress this aspect of the Gcd^−^ substitutions. Supporting this prediction, combining αE Gcn^−^ substitution E379K with each of the αI Gcd^−^ substitutions reduced the proportions of Gcn2 that coimmunoprecipitated with HA-LexA-KD (normalized P∶I ratios) from the elevated values seen for the Gcd^−^ single mutants to the lower values given by the Gcn^−^ single mutants (Fig. 7A–B). Similar results were observed on combining Gcn^−^ (R528A) and Gcd^−^ substitutions (T518A) in αI (Fig. 7A–B), supporting the idea that tighter association between the YKD and authentic KD contributes to the activating effect of Gcd^−^ substitutions in the αI segment of Gcn2.

**Figure 8:**
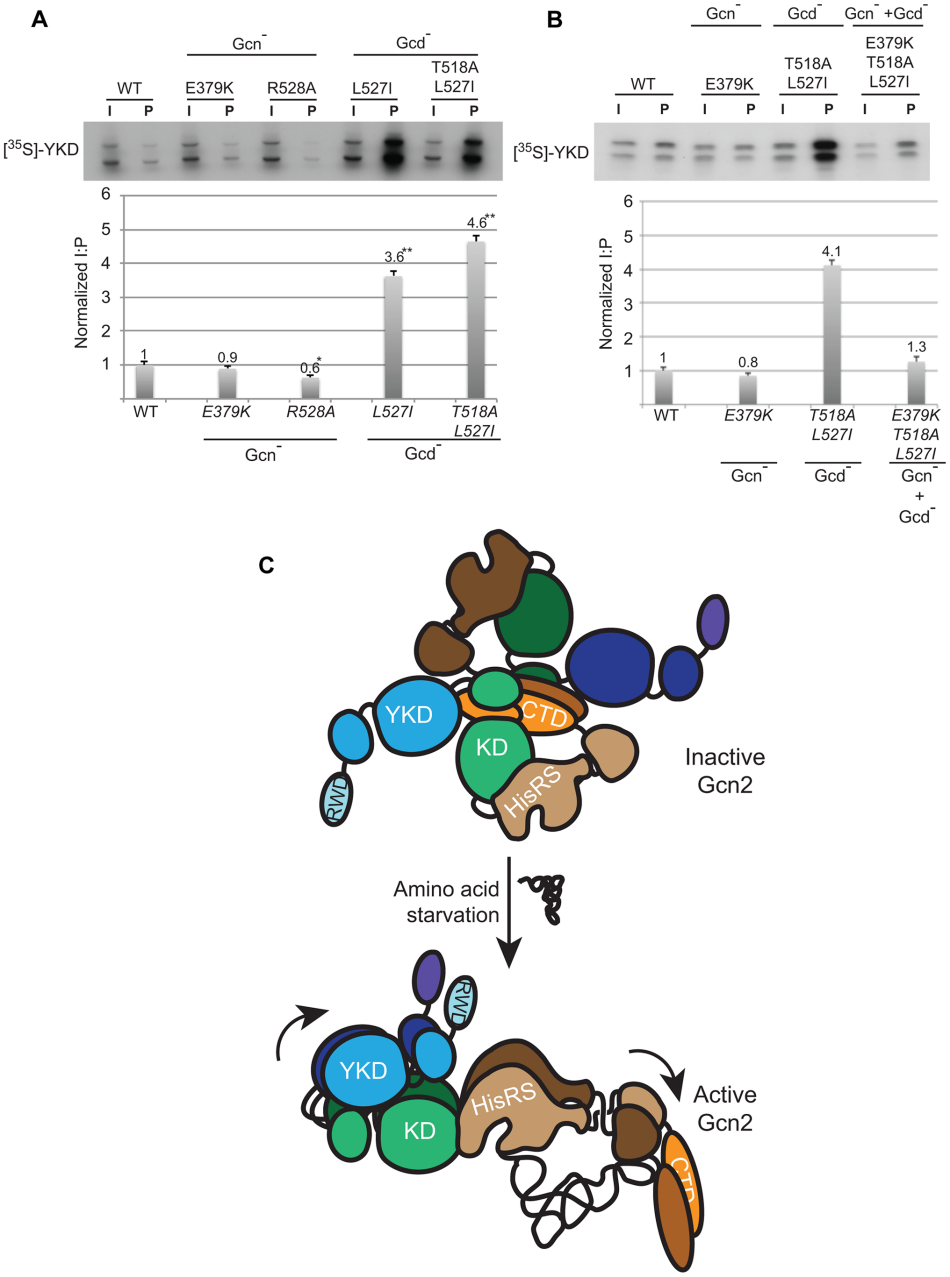
Gcd^−^ YKD substitutions in αI enhance direct binding between YKD and KD segments in a manner suppressed by Gcn^−^ YKD substitutions in αE. (**A–B**) YKD Gcn2 segments (WT or the indicated mutants) were translated *in vitro* with [^35^S]methionine and incubated with 50 µg of WCEs from transformants of *gcn2*Δ strain HQY132 expressing LexA-HA-KD(720–999). Reactions were immunoprecipitated with anti-HA antibodies and immune complexes were resolved by SDS-PAGE and visualized by fluorography. Input (I) lanes contain aliquots of reticulocyte lysates containing the input [^35^S]-labeled YKD fragments and pellet (P) lanes contain immune complexes recovered from reaction aliquots corresponding to 1/2 of the starting lysates. (**B**) Densities of bands in I and P lanes of (A) were quantified using a PhosphorImager Storm Scanner and ImageQuant software and P∶I ratios were calculated and normalized to that determined for the WT YKD fragment. Normalized ratios were calculated from three independent experiments and the average and S.E.M.s were plotted (**C**) Model of allosteric activation of the KD via direct interaction with the YKD, regulated by competing CTD∶KD association and tRNA binding to the HisRS-like domain. Inactive WT Gcn2 in nonstarved cells contains the CTD and HisRS-like domains engaged with the KD. These interactions could contribute directly to latency of the KD, but we propose here that the CTD acts indirectly to block the stimulatory YKD-KD interaction. YKD-CTD interaction helps to stabilize the inhibitory CTD-KD association in the inactive state. Uncharged tRNA binding to the HisRS domain and possibly also the CTD [14] would activate Gcn2 by dissociating the CTD from the KD to enable the stimulatory YKD-KD interaction uncovered in this study. YKD Gcn^−^ and Gcd- substitutions would weaken or strengthen, respectively, the stimulatory YKD-KD interaction.

We sought next to provide evidence that Gcd^−^ substitutions in αI increase a direct interaction between the YKD and KD. To this end, we incubated [^35^S]-methionine-labeled WT or mutant YKD fragments, synthesized by in vitro transcription/translation, with yeast extracts from a *gcn2*Δ strain containing the HA-LexA-KD fusion described above and measured the amounts of labeled YKD fragments that coimmunoprecipitated from the extracts with HA-LexA-KD. Interestingly, the L527I and T518A/L527I YKD fragments, harboring Gcd^−^ substitutions in αI, were co-immunoprecipitated with HA-LexA-KD at levels ∼4-fold higher than that observed for WT YKD (Fig. 8A). By contrast, the R528A YKD fragment, harboring a Gcn^−^ substitution in αI coimmunoprecipitated with HA-LexA-KD at only 60% of the WT value (Fig. 8A). Importantly, Gcn^−^ substitution E379K abolished the effect of the double Gcd^−^ substitutions T518A/L527I in the relevant triple mutant to render a level of YKD fragment binding only slightly higher than that given by E379K alone (Fig. 8B). These findings strongly support the notion that Gcd^−^ substitutions in αI increase the affinity of the YKD for the KD of Gcn2, whereas Gcn^−^ substitutions in αE or αI suppress this tighter interaction.

## Discussion

In this report, we provide strong evidence that the Gcn2 YKD is a positive regulatory domain required to overcome the latency of the adjoining KD under amino acid starvation conditions, and we identified specific residues located in a discrete region of the YKD that are likely situated at the YKD-KD interface and modulate, positively or negatively, this regulatory interaction. Mutation of certain residues at this predicted interface greatly impairs or eliminates phosphorylation of eIF2α on Ser-51 and confers the expected strong sensitivity to an inhibitor of histidine biosynthesis (3-AT) that signifies decreased induction of *GCN4* and its target amino acid biosynthetic genes. These Gcn^−^ mutations were obtained by targeting a subset of residues found to be evolutionarily conserved among YKDs of fungal Gcn2 homologs and that were predicted to reside on the surface of the YKD. These latter predictions were based on a sequence alignment of fungal Gcn2 YKDs with a group of authentic KDs, which allowed us to project onto the crystal structure of the yeast Gcn2 KD the sequence conservation of all YKD residues that can be aligned with residues in authentic KDs. According to the predicted tertiary structure of the YKD (Fig. 2), the substitutions found to have Gcn^−^ phenotypes alter residues that are visible on one face of the predicted YKD (Fig. 3C, middle image) and, hence, could define a single, continuous regulatory surface comprised of residues near helix αC, within the activation loop, and belonging to predicted helices αE and αI. As discussed below, however, substitutions in αC and the activation loop might identify an interaction site(s) distinct from that defined by Gcn^−^ substitutions in αE and αI of the YKD.

We also identified Gcd^−^ substitutions in the YKD, which increase eIF2α phosphorylation and derepress expression of the Gcn4-dependent *HIS4-lacZ* reporter in nonstarvation conditions. It is intriguing that the most potent of these substitutions also map in predicted αI, and that substitutions of adjacent residues in αI were identified that either impair (Gcn^−^) or constitutively activate (Gcd^−^) Gcn2 kinase function. These results identify αI as a key regulatory element of the YKD. Another group of Gcd^−^ substitutions alter residues predicted to reside in the hinge region of the YKD, which align closely with residues in the authentic Gcn2 KD that interact with one another and promote hinge rigidity in a manner believed to promote latency of the KD by restricting inter-lobe mobility [9]. Thus, inter-lobe mobility might also be important for the regulatory interactions of the YKD.

Of course, knowledge of the true locations of the YKD residues altered to produce Gcn^−^ or Gcd^−^ phenotypes will require structural analysis of this domain. However, the fact that the YKD structural model was instrumental in identifying functionally important conserved residues, at least some of which alter physical interaction between the YKD and KD, supports the idea that the affected amino acids define an important regulatory interface between the YKD and KD of Gcn2.

Previous work from our laboratory on Gcn2 derivatives lacking the entire YKD indicated that the YKD is not required for ribosome-binding [21], dimerization by full-length Gcn2 [19], or interactions with positive effectors Gcn1/Gcn20 [26]. Given that the YKD also is not required for kinase function *per se* [32], it appears to mediate the stimulatory effect of uncharged tRNA that elevates eIF2α phosphorylation in starved cells. In addition, we showed here that neither Gcn^−^ nor Gcd^−^ substitutions in the YKD alter tRNA binding by purified Gcn2 in a manner that would explain the alterations in kinase activity conferred by these substitutions. Accordingly, we hypothesized that the YKD substitutions alter a physical interaction between the YKD and KD that allosterically activates kinase function in starved cells in response to increased occupancy of the HisRS-like domain by uncharged tRNA.

Supporting this model, we found that the Gcd^−^ substitutions T518A and L527I in predicted αI of the YKD produce additive increases in association of full-length Gcn2 with the isolated Gcn2 KD in a LexA fusion in yeast WCEs. We showed previously, and confirmed here, that eliminating the YKD reduces the ability of otherwise WT Gcn2 to coimmunoprecipitate with the LexA-KD fusion. Because eliminating the authentic KD from Gcn2 abolishes, and not merely reduces, its interaction with LexA-KD in this assay [19], we presume that LexA-KD dimerizes with the KD in full-length Gcn2 and that additional interaction of the YKD with the KD moiety of LexA-KD increases the stability of the Gcn2·LexA-KD complex. In this view, the Gcd^−^ substitutions T518A and L527I increase the yield of Gcn2·LexA-KD complexes by strengthening YKD-KD interaction, which suggests in turn that the ability of these Gcd^−^ substitutions to activate kinase function results from tighter YKD-KD association within Gcn2. Additional evidence supporting this conclusion came from our finding that combining the Gcd^−^ substitutions T518A and L527I with the Gcn^−^ substitutions E379K and R528A completely suppressed the Gcd^−^ phenotype of the former mutations, with the strong 3AT^S^/Gcn^−^ phenotype of the latter mutations being expressed in the double mutants. Importantly, the Gcn^−^ substitutions E379K/R528A also abolished the increased coimmunoprecipitation of Gcn2 with the LexA-KD conferred by the Gcd^−^ substitutions. The co-suppression of both phenotypes strongly supports a mechanistic linkage between increased interaction between Gcn2 and LexA-KD (signifying increased YKD-KD association) and elevated Gcn2 kinase function. Further bolstering this conclusion, we provided direct evidence that the YKD Gcd^−^ substitutions T518A and L527I enhance interaction of recombinant YKD with LexA-KD in complexes reconstituted in vitro, whereas the Gcn^−^ substitutions E379K and R528A masked the stabilizing effect of the Gcd^−^ substitutions on YKD-KD association in the E379K/T518A/L527I triple mutant.

It might seem puzzling that (i) the YKD Gcd^−^ substitutions had greater effects on YKD-KD interactions than did the Gcn^−^ substitutions, even though both categories of substitutions evoke strong changes in Gcn2 kinase function in vivo; whereas the Gcn^−^ substitutions did substantially affect YKD-KD interactions when examined in the presence of YKD Gcd^−^ substitutions (Figs. 7–8). One way to explain these findings is to propose that the Gcn^−^ substitutions weaken a tighter YKD-KD association that is normally established only during activation of full-length Gcn2 by uncharged tRNA in starved cells. The normal activation process could be disrupted in the artificial Gcn2·LexA-KD complexes formed in the assays of Fig. 7; and they cannot occur in the assays of direct YKD-KD interactions shown in Fig. 8 because the tRNA-binding HisRS domain is absent in those constructs. By contrast, because the Gcd^−^ substitutions bypass the normal activation mechanism and strengthen YKD-KD interactions constitutively, their effects can be observed in either assay; and they can be reversed by Gcn^−^ substitutions that weaken direct YKD-KD contacts in the Gcd^−^ Gcn^−^ YKD double mutants. The fact that the Gcd^−^ triple substitution of YKD hinge residues Y353F/G363F/D406A had only a small effect on direct interaction between the YKD and KD (Fig. 7A–B) might indicate that these residues are not present at the YKD-KD interface and act indirectly to alter the conformation of full-length Gcn2 in a way that increases access of the YKD to the KD. However, as this triple substitution has a weaker Gcd^−^ phenotype compared to those conferred by the αI Gcd^−^ substitutions (cf. Figs. 4C &5C), it might simply have a correspondingly smaller effect on direct YKD-KD association.

Together, our results suggest that the YKD interacts directly with the KD dependent on residues in helix αI to mediate allosteric activation of Gcn2 kinase function. If this interaction is restricted to starvation conditions, as suggested above, then it presumably depends on other conformational changes within Gcn2 triggered by binding uncharged tRNA to the HisRS-like domain. One interesting possibility is prompted by our previous evidence that the CTD interacts with the KD to inhibit kinase function in a manner overcome by tRNA binding to the HisRS domain [15]. The CTD could inhibit the KD, at least partially, by the indirect mechanism of blocking the proposed stimulatory YKD-KD interaction. Binding of uncharged tRNA to the HisRS-like domain would partly dissociate the HisRS/CTD module from the KD segment and provide the YKD with access to its binding site(s) in the KD for allosteric stimulation of kinase function (Fig. 8C).

Our identification of Gcn^−^ mutations in the predicted activation loop of the YKD, and of Gcd^−^ substitutions in the YKD hinge region, raise the possibility that the conformation of the YKD is altered during the activation process in a manner that stabilizes the stimulatory YKD-KD interaction. It is unclear, however, how tRNA binding to the HisRS region would trigger this hypothetical alteration of YKD conformation. Accordingly, the predicted activation loop and hinge region, being exposed on the YKD surface, might simply provide additional contact points for the KD or CTD rather than mediating a conformational rearrangement of the YKD. In this view, the αI-αE surface of the C-lobe and one or more YKD segments including residues in αC, the hinge and activation loop would make independent stimulatory contacts with the KD (Fig. 8C). A precedent for this idea of a multivalent YKD-KD interaction surface is provided by activation of kinase LKB1 by the YKD STRAD in a manner facilitated by the scaffold protein MO25. Substrate binding determinants and the activation loop of STRAD contact the N- and C- lobe of LKB1, while STRAD's αC anchors it to MO25, and MO25 stabilizes the active conformation of the LKB1 (nonphosphorylated) activation loop [35].

## Materials and Methods

### Computational methods

Multiple sequence alignments were generated using MUSCLE at http://www.ebi.ac.uk/Tools/msa/muscle/. ConSurf [36] and PyMOL [37] were used to obtain sequence conservation scores and generate the surface representation of sequence conservation on the crystal structure of the yeast Gcn2 KD (pdb: 1ZYC).

### Plasmids and strains

Plasmids employed are listed in Table 1. QuikChange site-directed mutagenesis (Stratagene) was used to generate the novel derivatives of plasmid p722 (pSL201-pSL242) and p630 (pSL301-pSL311) and *GCN2* was sequenced in its entirety for those alleles exhibiting significant Gcn^−^ or Gcd^−^ phenotypes. For Gcd^−^ mutations identified by random mutagenesis, p722 was subjected to error-prone PCR mutagenesis using the GeneMorph II kit (Stratagene) by using primer pairs PS-1 (5′-ATAGCAAATTTAGAGAAAGAGTTAG-3′) and PS-2 (5′-CTTAACAGCAGTCATCGGTTTTAC-3′). The BlpI -XhoI 1.4-kb *GCN2* fragment encoding the YKD was isolated from plasmid DNA prepared from a pool of *E. coli* transformants harboring mutagenized plasmids and subcloned into p722. Plasmid DNA prepared from a pool of the resulting *E. coli* transformants was introduced into yeast strain H1149 and transformants were selected on minimal (SD) medium containing 0.5 mM 5-FT. Resident plasmids were isolated from colony-purified transformants and subjected to DNA sequence analysis to identify the mutations. As multiple mutations generally occurred, site-directed mutagenesis was used to produce plasmids pSL233, pSL234, pSL235, pSL237, pSL238, pSL239 and pSL240, containing only single mutations in *GCN2*. pSL102-pSL106 were generated by replacing the 1.2-kb BlpI-BspEI fragment encoding the YKD in pSL101 with the corresponding fragment from p722 derivatives harboring the appropriate *GCN2* mutations. The same strategy was used to construct pSL401-pSL405 from pHQ539.

**Table 1:** Plasmids used in this study.

Name	Description	Source or reference
p722	*CEN6 URA3 GCN2*	[32]
p2201	*gcn2-m2* in p722 backbone	[12]
p912	*gcn2-M788V* in p722 backbone	[33]
pSL201	*gcn2-E307P* in p722 backbone	This study
pSL202	*gcn2-R371A* in p722 backbone	This study
pSL203	*gcn2-L377K* in p722 backbone	This study
pSL204	*gcn2-L378K* in p722 backbone	This study
pSL205	*gcn2-E379K* in p722 backbone	This study
pSL206	*gcn2-P448L/E449L* in p722 backbone	This study
pSL207	*gcn2-L521K* in p722 backbone	This study
pSL208	*gcn2-F526K* in p722 backbone	This study
pSL209	*gcn2-R528A* in p722 backbone	This study
pSL210	*gcn2-K300E* in p722 backbone	This study
pSL211	*gcn2-L310A* in p722 backbone	This study
pSL212	*gcn2-E311A* in p722 backbone	This study
pSL213	*gcn2-T312A* in p722 backbone	This study
pSL214	*gcn2-L314A* in p722 backbone	This study
pSL215	*gcn2-H318A/V321A* in p722 backbone	This study
pSL216	*gcn2-I343A* in p722 backbone	This study
pSL217	*gcn2-L346A* in p722 backbone	This study
pSL218	*gcn2-E348A* in p722 backbone	This study
pSL219	*gcn2-W373A* in p722 backbone	This study
pSL220	*gcn2-H385A* in p722 backbone	This study
pSL221	*gcn2-H391A* in p722 backbone	This study
pSL222	*gcn2-K392A* in p722 backbone	This study
pSL223	*gcn2-K413A* in p722 backbone	This study
pSL224	*gcn2-V423A* in p722 backbone	This study
pSL225	*gcn2-W445A* in p722 backbone	This study
pSL226	*gcn2-T462A* in p722 backbone	This study
pSL227	*gcn2-D463A* in p722 backbone	This study
pSL228	*gcn2-W465A* in p722 backbone	This study
pSL229	*gcn2-G468A* in p722 backbone	This study
pSL230	*gcn2-D502A/L503K* in p722 backbone	This study
pSL231	*gcn2-K513A/K514A/R515A* in p722 backbone	This study
pSL232	*gcn2-R515A* in p722 backbone	This study
pSL233	*gcn2-D497Y* in p722 backbone	This study
pSL234	*gcn2-T518A* in p722 backbone	This study
pSL235	*gcn2-L527I* in p722 backbone	This study
pSL236	*gcn2-T518A/L527I* in p722 backbone	This study
pSL237	*gcn2-N530K* in p722 backbone	This study
pSL238	*gcn2-Y353F* in p722 backbone	This study
pSL239	*gcn2-G363F* in p722 backbone	This study
pSL240	*gcn2-D406A* in p722 backbone	This study
pSL241	*gcn2-G363F/D406A* in p722 backbone	This study
pSL242	*gcn2-Y353F/G363F/D406A* in p722 backbone	This study
pSL101	*P_GAL_-FLAG-TEV-GCN2*	[23]
pSL102	*P_GAL_-FLAG-TEV-gcn2-m2* in pSL101 backbone	This study
pSL103	*P_GAL_-FLAG-TEV-gcn2-E379K* in pSL101 backbone	This study
pSL104	*P_GAL_-FLAG-TEV*-*gcn2-R528A* in pSL101 backbone	This study
pSL105	*P_GAL_-FLAG-TEV-gcn2-L527I* in pSL101 backbone	This study
pSL106	*P_GAL_-FLAG-TEV-gcn2-Y353F/G363F/D406A* in pSL101 backbone	This study
p630	*URA3* 2-micron *GCN2*	[32]
p2327	*gcn2-Δ324-538* in p630 backbone	[19]
pSL301	*gcn2-E307P* in p630 backbone	This study
pSL302	*gcn2-E379K* in p630 backbone	This study
pSL303	*gcn2-P448L/E449L* in p630 backbone	This study
pSL304	*gcn2-R528A* in p630 backbone	This study
pSL305	*gcn2-T518A* in p630 backbone	This study
pSL306	*gcn2-L527I* in p630 backbone	This study
pSL307	*gcn2-T518A/L527I* in p630 backbone	This study
pSL308	*gcn2-Y353F/G363F/D406A* in p630 backbone	This study
pSL309	*gcn2-T518A/E379K* in p630 backbone	This study
pSL310	*gcn2-T518A/R528A* in p630 backbone	This study
pSL311	*gcn2-L527I/E379K* in p630 backbone	This study
pHQ539	*GCN2 (230–604)* under T7 promotor	[19]
pSL401	*gcn2-E379K* in pHQ539 backbone	This study
pSL402	*gcn2-R528A* in pHQ539 backbone	This study
pSL403	*gcn2-L527I* in pHQ539 backbone	This study
pSL404	*gcn2-T518A/L527I* in pHQ539 backbone	This study
pSL405	*gcn2-E379K/T518A/L527I* in pHQ539 backbone	This study
pHQ587	*HIS3* 2-micron *P_GAL_-LexA-HA-GCN2 (720–999)*	[19]

Yeast strains employed included H1149 (*MATα gcn2*Δ*::LEU2 ino1 ura3-52 leu2-3 leu2-112 <HIS4-lacZ>*) [11], HQY132 (*MAT*α *trp1 ura3 his3 lexAop-LEU2 gcn2*Δ*::hisG*) [19], and H2684 (*MATa ino1 ura3-52 gcn1*Δ *gcn2*Δ *gcn20*Δ) (M. Marton and A.G.H., unpublished observations).

### Protein purification

Transformants of H2684 bearing plasmids pSL101, pSL102, pSL103, pSL104, pSL105, or pSL106 were grown to saturation in SC-Ura medium, diluted to A_600_ = 0.2 in SC-Ura containing 10% galactose as carbon source and grown to A_600_ ∼2.5. Cells were harvested (∼25 g), washed with cold distilled water containing EDTA-free protease inhibitor cocktail (PIC) (Boehringer Mannheim) and 0.5 mM PMSF, resuspended in ice-cold binding buffer (BB) (100 mM sodium phosphate [pH 7.4], 500 mM NaCl, 0.1% Triton X-100, EDTA-free PIC, 1 µg/ml leupeptin, and 1 mM PMSF) and disrupted using SPEX freezer mill (model *6870*). Lysates were clarified by centrifugation at 39,000×g for 2 h at 4°C and mixed with 1 ml of M2-FLAG affinity resin (Sigma) overnight at 4°C. The resin was washed three times with 10 vol of BB and Gcn2 was eluted with 100 units of AcTEV protease in 500 µl of 1× TEV buffer (50 mM Tris pH 8, 0.5 mM EDTA, 1 mM DTT). The eluates were concentrated with an Amicon Centricon filter (exclusion limit of M_r_ 10,000) and dialyzed against 10 mM Tris-HCl [pH 7.4], 50 mM NaCl, 20% glycerol and stored at −80°C. The eIF2α−ΔC protein was purified from *E. coli* as previously described [13].

### Biochemical assays in Whole Cell Extracts (WCEs)

Assays of β-galactosidase activity in WCEs were performed as described previously [38]. For Western analysis, WCEs were prepared by trichloroacetic acid extraction, as described previously [39], and immunoblot analysis was conducted as described [19] using phosphospecific antibodies against eIF2α-P (Biosource International) and polyclonal antibodies against eIF2α [40] or Gcn2 [16].

### Kinase and tRNA-binding assays of purified Gcn2

Assaying autophosphorylation and eIF2α phosphorylation by purified Gcn2 was conducted as described previously [8]. Binding of tRNA was measured with a gel mobility shift assay as follows. Total yeast tRNA was purchased from Roche. tRNA was first dephosphorylated using calf intestine alkaline phosphatase (New England BioLabs) for 1 h at 37°C in 1× Dephosphorylation buffer provided with the enzyme and followed by phenol/chloroform extraction and ethanol precipitation. Three micrograms of the dephosphorylated tRNA was phosphorylated using 25 pmol of [γ-^32^P]ATP and 10 units polynucleotide kinase for 1 h in 1× kinase buffer provided with the enzyme. The reaction was stopped by adding EDTA to 1 mM and heating for 2 min at 95°C. tRNA was purified from free nucleotides using MicroSpin G-25 Columns (GE Healthcare). Fifty nanograms of [^32^P]-labelled tRNA were mixed with purified Gcn2 (1–4 µM) and 10 U RNasin Ribonuclease Inhibitor (N2511; Promega), in 1× GMSA buffer (2 mM HEPES [pH 7.4], 15 mM NaCl, 15 mM MgCl_2_, 10% glycerol) in a total volume of 20 µl. After incubating for 30 min at 30°C, 4 µl of 6× nucleic acid loading buffer (30% (v/v) glycerol, 0.25% (w/v) bromophenol blue) were added and the mixture was resolved by electrophoresis on a 1% agarose gel cast in 1× MOPS buffer (Quality Biological, Inc) at 100 V for 1.5 h. RNA and protein molecules were transferred from the gel to a nitrocellulose membrane (162-0097; Bio-Rad, Hercules, CA, USA) by capillary action in 10× SSC for 16 h. [^32^P]tRNA–Gcn2 complexes were quantified with a phosphorimager (Molecular Dynamics) using the Image Quant software provided by the vendor.

### Co-immunoprecipitation and immunoblotting

Coimmunoprecipitations of Gcn2 with LexA-HA-KD fusion protein were conducted as described previously [19] using HA-probe Antibody agarose conjugate (sc-7392 AC) and LexA antibodies (sc-7544, 1∶2000 dilution) purchased from Santa Cruz. Coimmunoprecipitation of [^35^S]-methionine-labeled Gcn2 YKD polypeptides with LexA-HA-KD fusion protein was executed as follows. *In vitro* transcription/translation with [^35^S]-methionine was conducted using the TNT T7 Coupled Reticulocyte Lysate System (Promega) according to the vendor's instructions. *In vitro*-translated proteins were partially purified by ammonium sulfate precipitation as described previously [41] and resuspended in 50 µl of buffer A (20 mM Tris/HCl pH 7.5, 100 mM NaCl, 0.2 mM EDTA, 1 mM DTT) containing 12.5% glycerol. Fifty µg of WCE prepared from p2825 transformants of HQY132 and 10 µL of *in vitro*-translated proteins were diluted to a final volume of 200 µL with breaking buffer (50 mM Tris/HCl, ph 7.5, 50 mM NaCl, 0.1% Triton-100, 1 mM DTT) containing protease inhibitors (Aprotinin 10 µg/mL, Leupeptin 10 µg/mL, Pepstatin 10 µg/mL and 1 mM PMSF) and pre-incubated with 20 µL of protein A-agarose beads (Santa Cruz, sc-2001) suspended in breaking buffer for 1 h at 4°C with rocking. The beads were removed by centrifugation and the supernatant added to HA-probe Antibody agarose conjugate (Santa Cruz, sc-7392) and incubated at 4°C for 2 h with rocking. The beads were collected by centrifugation, washed three times with 500 µl of breaking buffer, and resuspended in 40 µl of Tris-Glycine SDS Sample Buffer (Novex). Proteins in the immune complexes were resolved by sodium dodecyl sulfate (SDS)-polyacrylamide gel electrophoresis (PAGE). For detecting [^35^S]-labeled proteins, gels were fixed with 25% ethanol/10% acetic acid, treated with Amplify (Amersham), dried, and subjected to fluorography.

## Supporting Information

Figure S1 Structure-based sequence alignment of the YKD region of fungal Gcn2 proteins with authentic KDs. (**A–G**) Multiple sequence alignment of Gcn2 YKDs from 29 fungal species, and KDs from 12 different eIF2α kinases and 15 other kinases, was built using the MUSCLE program, and amino acid residue coloring was generated with the software MacClade 4.08. Sequences are identified on the far left with abbreviations of their species of origin. Numbering corresponds to residue positions in full-length *S. cerevisiae* Gcn2 (residues 280–534). Regions of α-helical and β-strand secondary structures are denoted at the top based on their locations in the Gcn2 KD (pdb: 1ZYC), along with the positions of signature motifs critical for kinase function. Gcn2 YKD substitutions examined in this study are shown along the top at their positions in the alignment, with those conferring Gcn^−^ phenotypes shown in red, those conferring Gcd^−^ phenotypes shown in green, and those preserving WT function shown in black.
(PDF)

Figure S2 Summary of phenotypes conferred by targeted substitutions of residues highly conserved among fungal Gcn2 YKDs and predicted to be surface-exposed. (**A–B**) Transformants of *gcn2*Δ strain H1149 containing derivatives of low-copy *GCN2* plasmid p722 harboring known Gcn^−^ mutation *m2*, known Gcd^−^ mutation *M788V*, or the indicated mutations altering predicted segments of the Gcn2 YKD were replica-plated to SC-Ura, SC-Ura plus 30 mM 3-AT, or SD plus 0.5 mM 5-FT/0.125 mM TRA (5FT/TRA) and incubated for 3 d at 30°C. The predicted secondary structure elements of the YKD altered by the mutations are given schematically to the left of the allele names. Except for *H385A* (highlighted with an asterisk), which reduces Gcn2 protein abundance, none of these mutations altered sensitivity to 3-AT or 5-FT/TRA and, hence, do not appear to affect Gcn2 function. (**C**) Complete list of mutations examined in this study that alter the YKD. Growth on SC containing 3-AT or on SD containing 5-FT/TRA was examined as described in (A–B), and in Figs. 3A, 4A, and 5A, and is summarized qualitatively in columns 2 and 3, respectively. Column 4 summarizes the results of Western analysis of WCEs using antibodies against Gcn2 as described in Figs. 3B, 4B, and 5B and from data not shown. Only the *gcn2-H385A* product was found to be expressed at lower than WT levels, and was undetectable (data not shown).
(PDF)
