# Supplementary figures and images for: Enhanced Interaction between Pseudokinase and Kinase Domains in Gcn2 stimulates eIF2α Phosphorylation in Starved Cells
Source: PLoS Genet. 2014 May 8;10(5):e1004326. doi: 10.1371/journal.pgen.1004326 (PMC4014428; doi:10.1371/journal.pgen.1004326)

Fig.S1-A

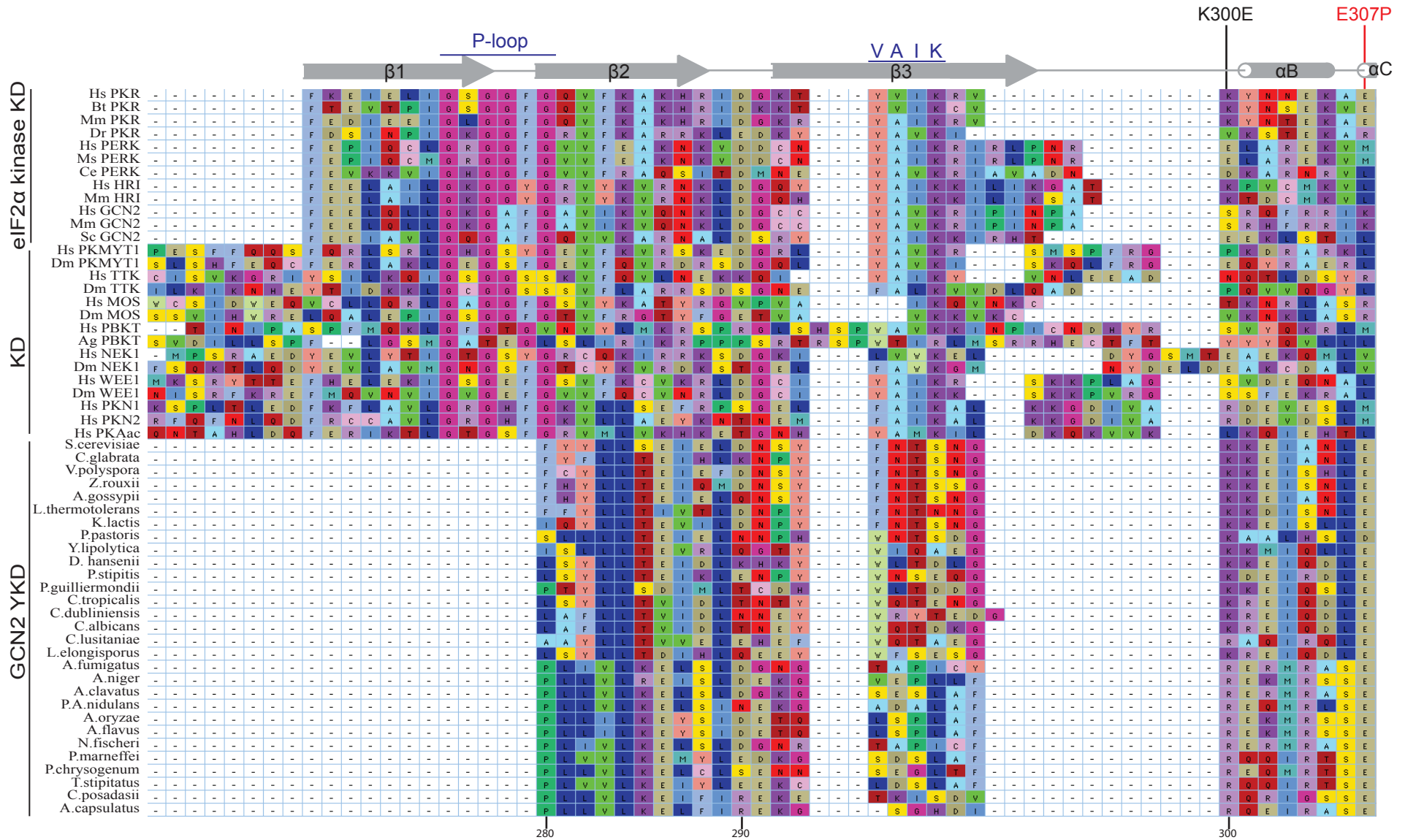

Fig.S1-B

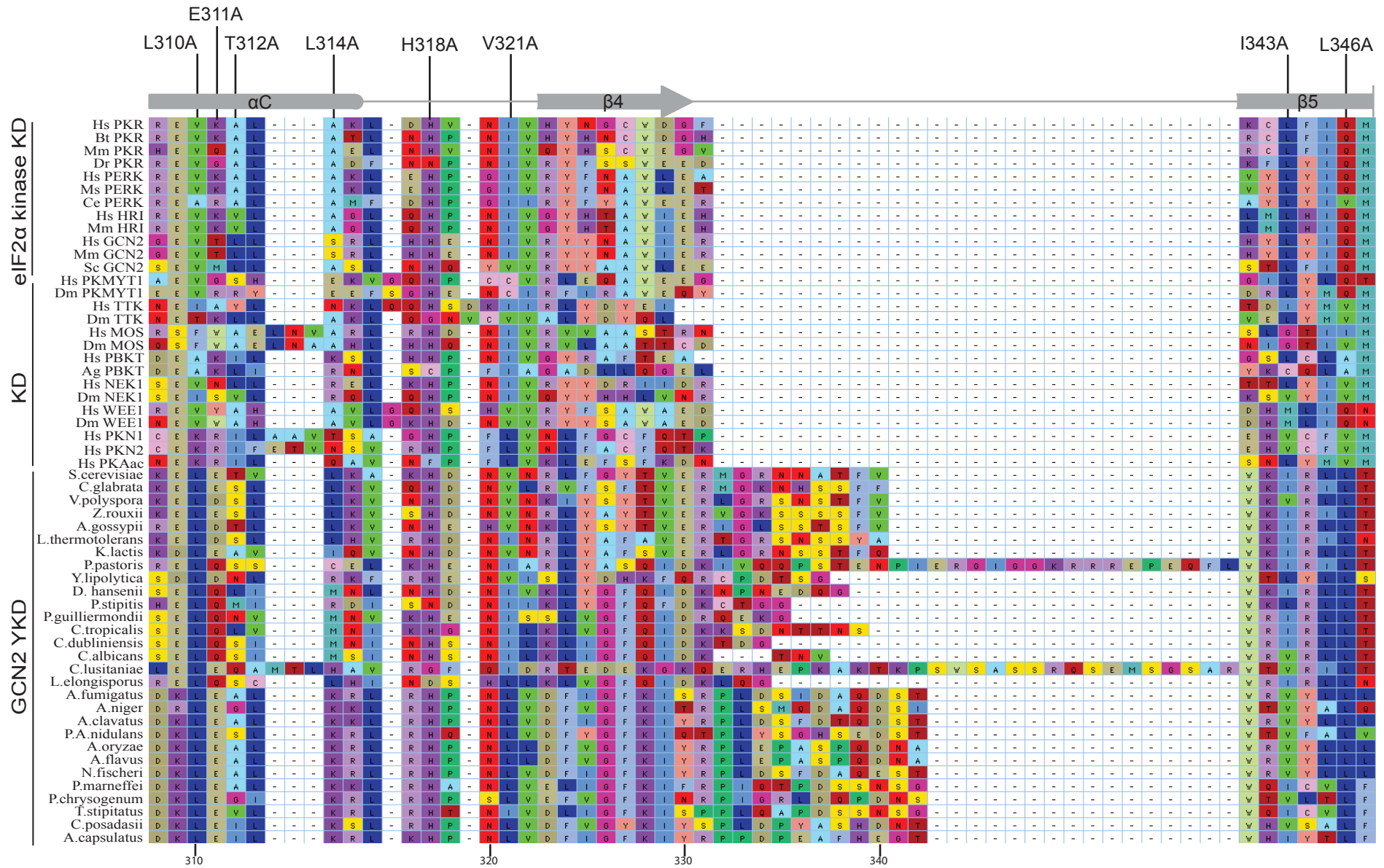



Fig.S1-D

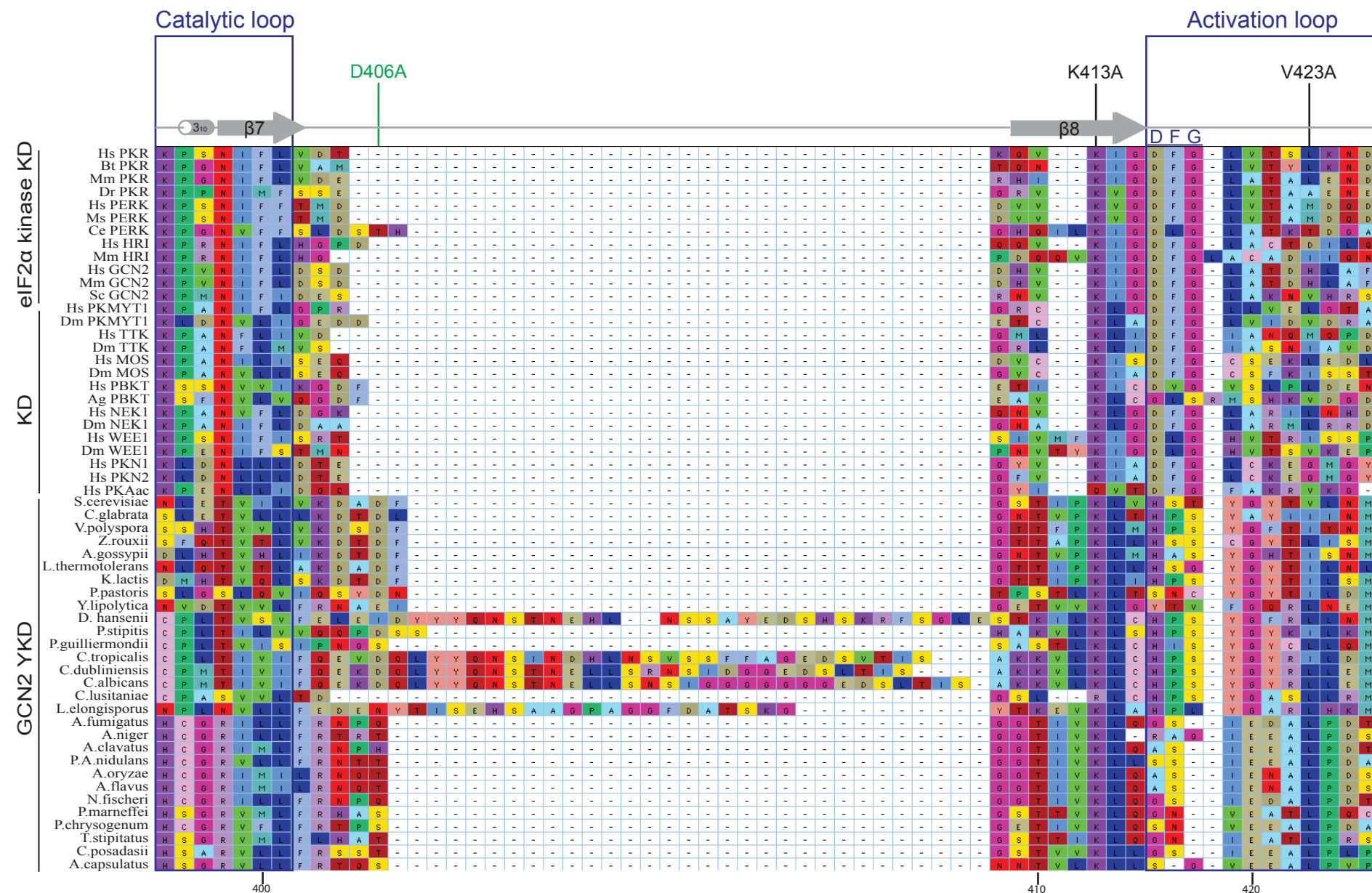

Fig.S1-E

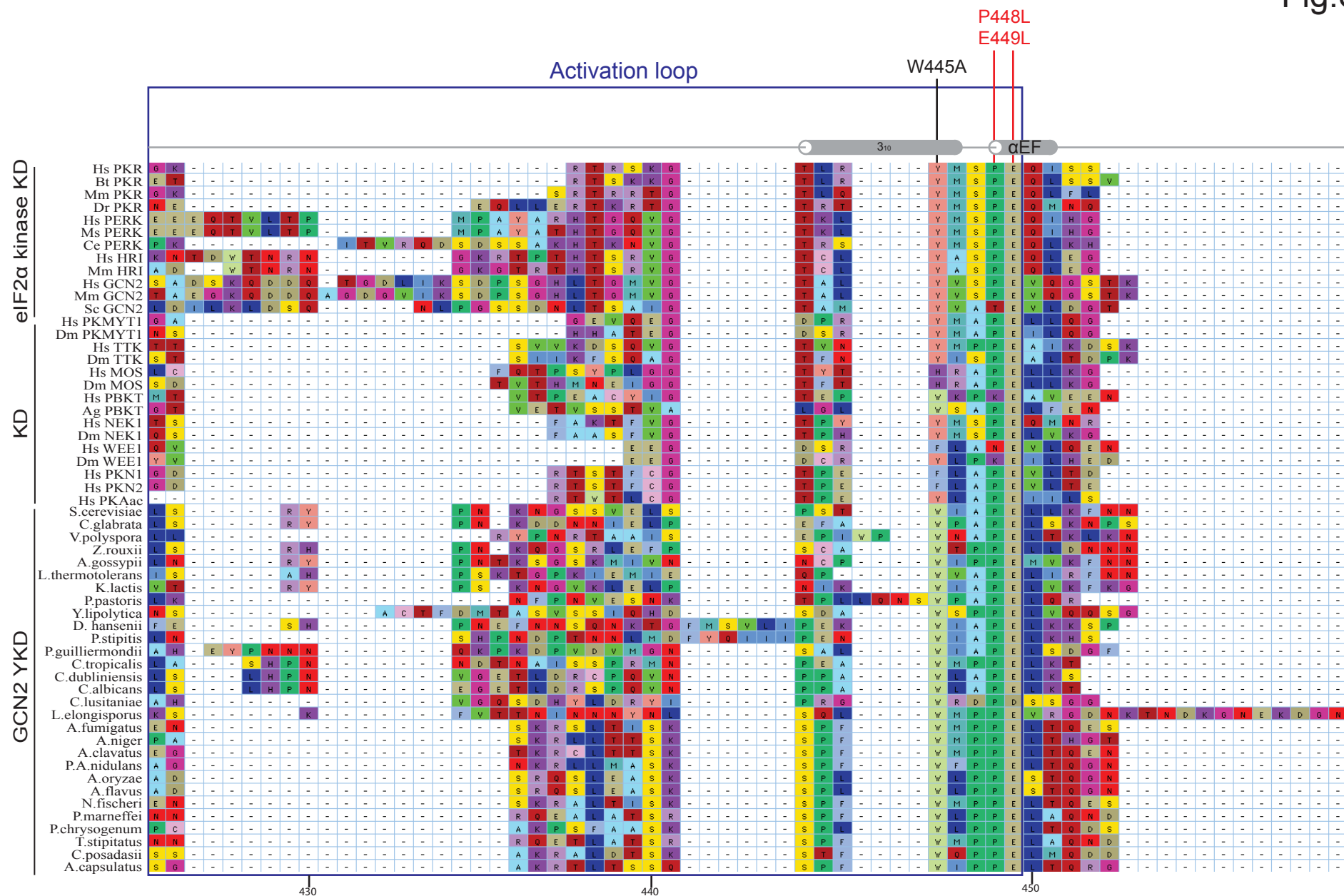

Fig.S1-F

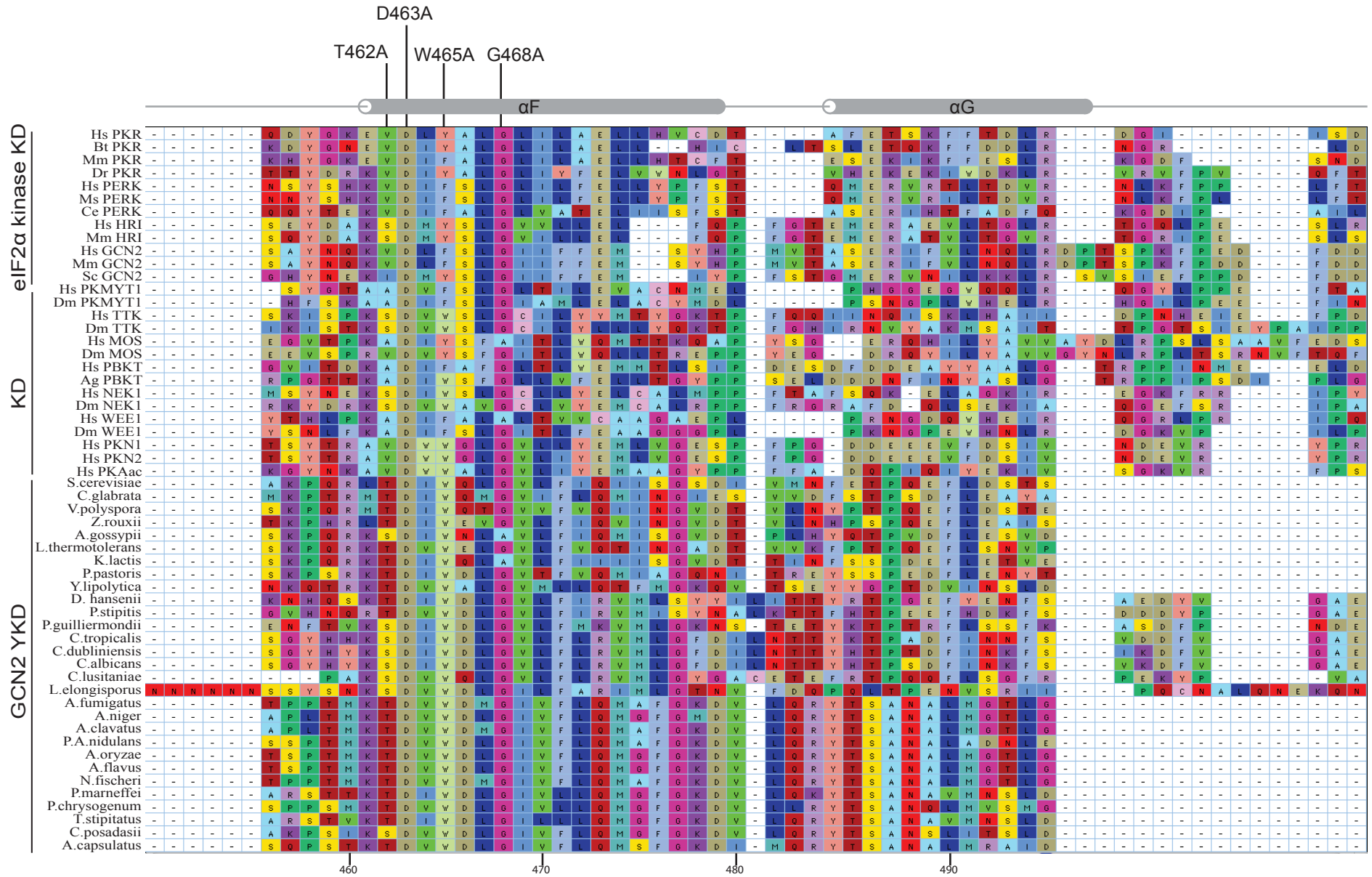

Fig.S1-G

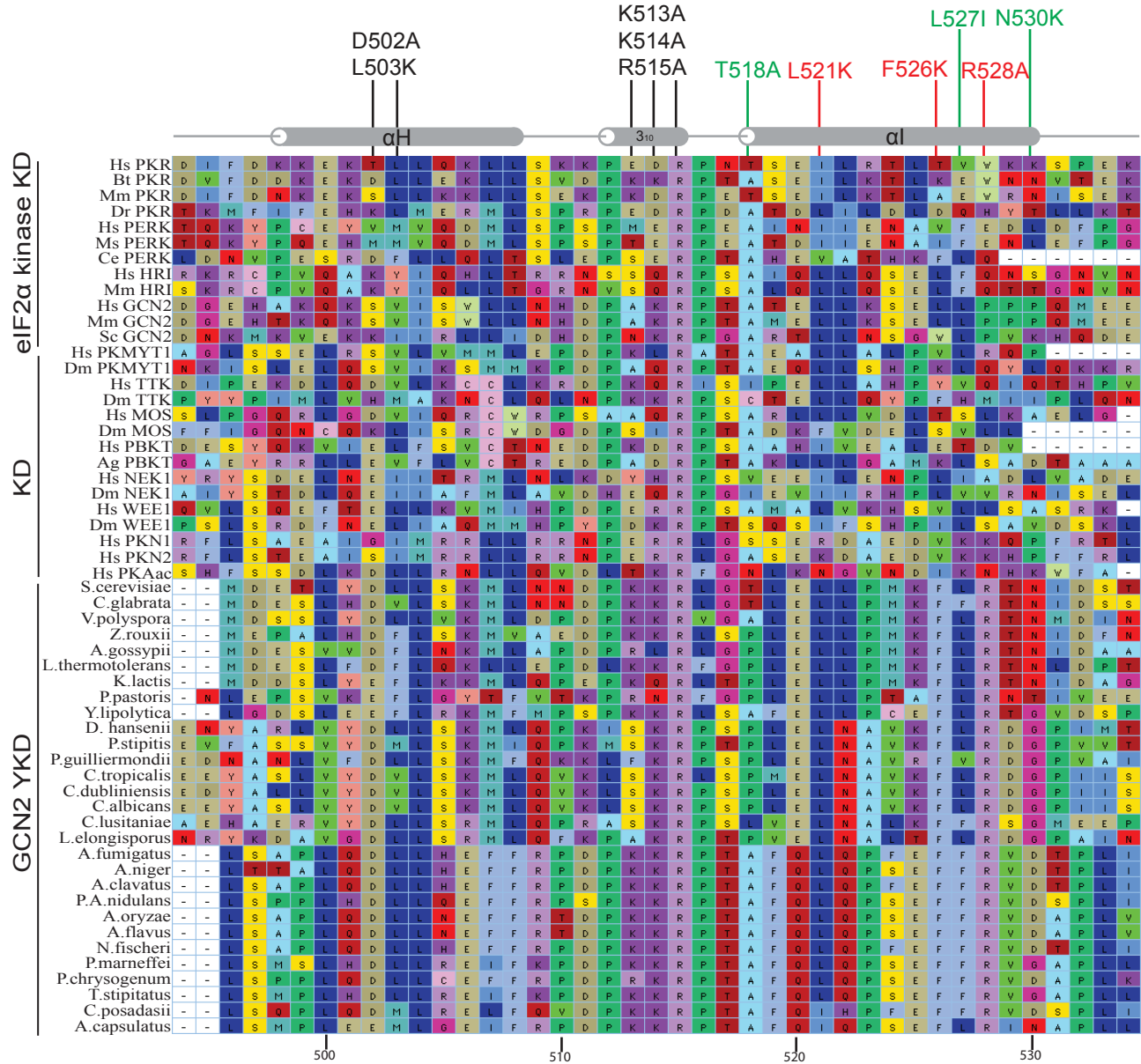

Supplement: Figure S1 — Structure-based sequence alignment of the YKD region of fungal Gcn2 proteins with authentic KDs. (A–G) Multiple sequence alignment of Gcn2 YKDs from 29 fungal species, and KDs from 12 different eIF2α kinases and 15 other kinases, was built using the MUSCLE program, and amino acid residue coloring was generated with the software MacClade 4.08. Sequences are identified on the far left with abbreviations of their species of origin. Numbering corresponds to residue positions in full-length S. cerevisiae Gcn2 (residues 280–534). Regions of α-helical and β-strand secondary structures are denoted at the top based on their locations in the Gcn2 KD (pdb: 1ZYC), along with the positions of signature motifs critical for kinase function. Gcn2 YKD substitutions examined in this study are shown along the top at their positions in the alignment, with those conferring Gcn− phenotypes shown in red, those conferring Gcd− phenotypes shown in green, and those preserving WT function shown in black. (PDF) [file pgen.1004326.s001.pdf]
