# Supplementary material for: Enhanced Interaction between Pseudokinase and Kinase Domains in Gcn2 stimulates eIF2α Phosphorylation in Starved Cells
Source: PLoS Genet. 2014 May 8;10(5):e1004326. doi: 10.1371/journal.pgen.1004326 (PMC4014428; doi:10.1371/journal.pgen.1004326)
Supplement: Figure S2 — Summary of phenotypes conferred by targeted substitutions of residues highly conserved among fungal Gcn2 YKDs and predicted to be surface-exposed. (A–B) Transformants of gcn2Δ strain H1149 containing derivatives of low-copy GCN2 plasmid p722 harboring known Gcn− mutation m2, known Gcd− mutation M788V, or the indicated mutations altering predicted segments of the Gcn2 YKD were replica-plated to SC-Ura, SC-Ura plus 30 mM 3-AT, or SD plus 0.5 mM 5-FT/0.125 mM TRA (5FT/TRA) and incubated for 3 d at 30°C. The predicted secondary structure elements of the YKD altered by the mutations are given schematically to the left of the allele names. Except for H385A (highlighted with an asterisk), which reduces Gcn2 protein abundance, none of these mutations altered sensitivity to 3-AT or 5-FT/TRA and, hence, do not appear to affect Gcn2 function. (C) Complete list of mutations examined in this study that alter the YKD. Growth on SC containing 3-AT or on SD containing 5-FT/TRA was examined as described in (A–B), and in Figs. 3A, 4A, and 5A, and is summarized qualitatively in columns 2 and 3, respectively. Column 4 summarizes the results of Western analysis of WCEs using antibodies against Gcn2 as described in Figs. 3B, 4B, and 5B and from data not shown. Only the gcn2-H385A product was found to be expressed at lower than WT levels, and was undetectable (data not shown). (PDF) [file pgen.1004326.s002.pdf]

Fig.S2

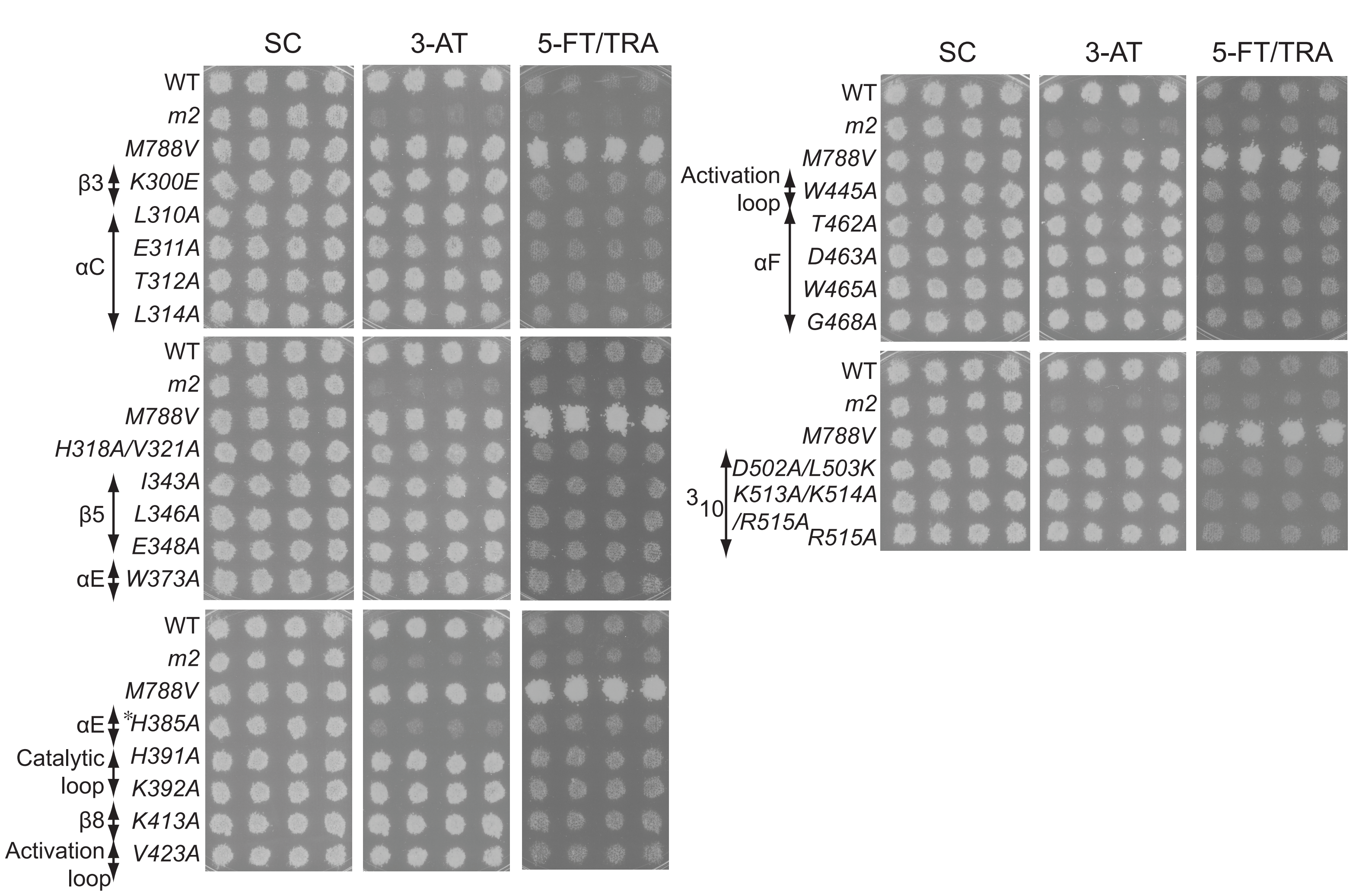

C

	GCN2 allele	Growth on 3-AT	Growth on 5-FT/TRA	Protein expression
	WT	+++	-	+++
(HisRS)	*m2*	-	-	+++
(Hinge)	*M788V*	+++	+++	+++
β3	*K300E*	+++	-	+++
αC	*E307P*	-	-	+++
αC	*L310A*	+++	-	+++
αC	*E311A*	+++	-	+++
αC	*T312A*	+++	-	+++
αC	*L314A*	+++	-	+++
	H318A/V321A	+++	-	+++
β5	*I343A*	+++	-	+++
β5	*L346A*	+++	-	+++
β5	*E348A*	+++	-	+++
Hinge	*Y353F*	+++	-/+	+++
Hinge	*G363F*	+++	-/+	+++
αE	*R371A*	+	-	+++
αE	*W373A*	+++	-	+++
αE	*L377K*	-	-	+++
αE	*L378K*	-	-	+++
αE	*E379K*	-	-	+++
αE	*H385A**	-	-	-
Catalytic loop	*H391A*	+++	-	+++
Catalytic loop	*K392A*	+++	-	+++
Hinge	*D406A*	+++	-	+++
Hinge	*G363F/D406A*	+++	+	+++
Hinge	*Y353F/G363F/D406A*	+++	+++	+++
β8	*K413A*	+++	-	+++
Activation loop	*V323A*	+++	-	+++
Activation loop	*W445A*	+++	-	+++
Activation loop	*P448L/E449L*	-	-	+++
αF	*T462A*	+++	-	+++
αF	*D465A*	+++	-	+++
αF	*G468A*	+++	-	+++
	D497Y	+++	-	+++
3_{10}	*D502A/L503K*	+++	-	+++
3_{10}	*K513A/K514A/R515A*	+++	-	+++
3_{10}	*R515A*	+++	-	+++
αI	*T518A*	+++	-/+	+++
αI	*L521K*	-	-	+++
αI	*F526K*	-	-	+++
αI	*L527I*	+++	-	+++
αI	*T518A/L527I*	+++	+++	+++
αI	*R528A*	-	-	+++
αI	*N530K*	+++	++	+++
